# Different modification pathways for m^1^A58 incorporation in yeast elongator and initiator tRNAs

**DOI:** 10.1093/nar/gkad722

**Published:** 2023-08-31

**Authors:** Marcel-Joseph Yared, Yasemin Yoluç, Marjorie Catala, Carine Tisné, Stefanie Kaiser, Pierre Barraud

**Affiliations:** Expression génétique microbienne, Université Paris Cité, CNRS, Institut de biologie physico-chimique, Paris, France; Department of Chemistry, Ludwig Maximilians University, Munich, Germany; Expression génétique microbienne, Université Paris Cité, CNRS, Institut de biologie physico-chimique, Paris, France; Expression génétique microbienne, Université Paris Cité, CNRS, Institut de biologie physico-chimique, Paris, France; Department of Chemistry, Ludwig Maximilians University, Munich, Germany; Institute of Pharmaceutical Chemistry, Goethe-University, Frankfurt, Germany; Expression génétique microbienne, Université Paris Cité, CNRS, Institut de biologie physico-chimique, Paris, France

## Abstract

As essential components of the protein synthesis machinery, tRNAs undergo a tightly controlled biogenesis process, which include the incorporation of numerous posttranscriptional modifications. Defects in these tRNA maturation steps may lead to the degradation of hypomodified tRNAs by the rapid tRNA decay (RTD) and nuclear surveillance pathways. We previously identified m^1^A58 as a late modification introduced after modifications Ψ55 and T54 in yeast elongator tRNA^Phe^. However, previous reports suggested that m^1^A58 is introduced early during the tRNA modification process, in particular on primary transcripts of initiator tRNA_i_^Met^, which prevents its degradation by RNA decay pathways. Here, aiming to reconcile this apparent inconsistency on the temporality of m^1^A58 incorporation, we examined its introduction into yeast elongator and initiator tRNAs. We used specifically modified tRNAs to report on the molecular aspects controlling the Ψ55 → T54 → m^1^A58 modification circuit in elongator tRNAs. We also show that m^1^A58 is efficiently introduced on unmodified tRNA_i_^Met^, and does not depend on prior modifications. Finally, we show that m^1^A58 has major effects on the structural properties of initiator tRNA_i_^Met^, so that the tRNA elbow structure is only properly assembled when this modification is present. This observation provides a structural explanation for the degradation of hypomodified tRNA_i_^Met^ lacking m^1^A58 by the nuclear surveillance and RTD pathways.

## Graphical Abstract

**Graphical Abstract:**
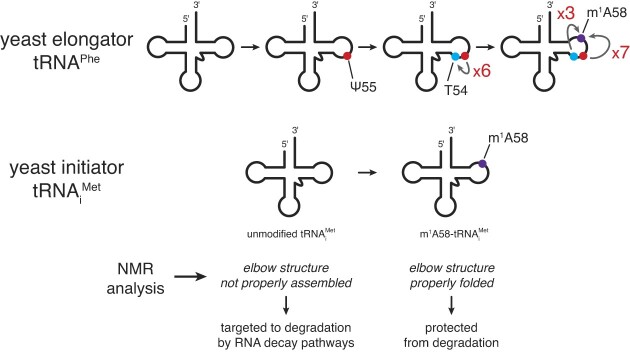

## INTRODUCTION

Transfer RNAs (tRNAs) are essential components of the cellular protein synthesis machinery, but also serve additional functions outside translation (1–4). To achieve their wide range of functions within cells, tRNAs undergo a tightly controlled biogenesis process leading to the formation of mature tRNAs (5–8). The biogenesis of tRNAs typically includes the removal of the 5′-leader and 3′-trailer sequences from the precursor-tRNA transcripts, the addition of the 3′-CCA amino-acid accepting sequence, and the incorporation of a large number of posttranscriptional chemical modifications. These modifications occur at specific sites in a tightly controlled manner, which ensures that the tRNA biogenesis process effectively leads to the formation of functional tRNAs (9–13). All the cellular functions of tRNAs are, to various extents, affected by modifications. In particular, modifications in and around the anticodon are implicated in the decoding process (9,14–17), whereas modifications found in the tRNA core are collectively implicated in the folding and stability of tRNAs (18–21). Posttranscriptional modifications are thus central to tRNA biology. Maturation defects, resulting in lack of modifications in the tRNA core, may result in alternative folding (22,23), and often reduce tRNA stability, leading to the degradation of hypomodified tRNAs by the rapid tRNA decay (RTD) pathway (24–26) and the nuclear surveillance pathway (27–29).

Although modifications are typically introduced in tRNAs independently of each other, several modification circuits have been identified in which one or more modifications stimulate or repress the incorporation of another modification (11,30,31). This obviously drives a defined sequential order in the tRNA modification process. Most of the reported examples of an ordered modification process occur in the tRNA anticodon loop region (32–36), but modification circuits in the tRNA core have also been reported (37–39).

One such circuit in the tRNA core involves modifications in the T-loop of yeast tRNAs. Using NMR spectroscopy to monitor the maturation of tRNAs in a time-resolved fashion in yeast extracts (40), we previously identified a sequential order in the introduction of T54, Ψ55 and m^1^A58 in yeast tRNA^Phe^, with Ψ55 being introduced first, then T54 and finally m^1^A58 (39). Using specific deletion strains, we uncovered a cross-talk between these three modifications, with the m^1^A58 modification strongly dependent on the two others. In a *pus4Δ* strain, lacking Ψ55, we indeed observed a severe slow-down in the introduction of both T54 and m^1^A58. Similarly, in a *trm2Δ* strain, lacking T54, we observed a slow-down in the introduction of m^1^A58 (39). In addition, we showed, using liquid-chromatography coupled with tandem mass spectrometry (LC-MS/MS), that levels of m^1^A58 and T54 are affected in the *pus4Δ* and *trm2Δ* strains, in both yeast tRNA^Phe^ and in total yeast tRNAs, in a manner compatible with the cross-talk observed with NMR spectroscopy in yeast extracts. This demonstrated that these cross-talks in the T-loop are manifest not only in tRNA^Phe^ but also in other yeast tRNAs. Overall, the slow-down in the incorporation of modifications and the corresponding decrease in the modification levels observed in the absence of a specific enzyme, namely in the *pus4Δ* and *trm2Δ* strains, was interpreted as a positive effect of the corresponding modification on the introduction of the other ones. We thus concluded that two modification circuits exist in the T-loop of yeast tRNAs, the long-branch Ψ55 $ \to$ T54 $ \to$ m^1^A58 circuit and the direct-branch Ψ55 $ \to$ m^1^A58 circuit, without being able to conclude on the direct or indirect nature of the effect of Ψ55 on m^1^A58 (39).

Overall, this report on yeast tRNA^Phe^ identified m^1^A58 as a late modification, introduced after earlier modifications such as Ψ55, T54 and m^7^G46 (39). However, previous reports suggested that m^1^A58 is introduced early along the tRNA modification process in yeast, with m^1^A58 being introduced on initial pre-tRNA transcripts (5). Yeast initiator pre-tRNA_i_^Met^ lacking m^1^A58, but containing the 5′-leader and part of the 3′-trailer sequences, is targeted by the nuclear surveillance and RTD pathways (27,28,41,42). In yeast tRNA_i_^Met^, the m^1^A58 modification is part of an unusual tRNA elbow structure involving non-canonical nucleotides A20, A54 and A60. This unusual substructure is assembled via an intricate network of interactions between the D- and T-loops and is likely conserved in eukaryotic initiator tRNAs (43). Altogether, these reports led to the model that m^1^A58 is introduced on pre-tRNA_i_^Met^ transcripts, which stabilizes the tRNA_i_^Met^ unique substructure, thereby preventing its degradation. In addition, degradation of tRNA_i_^Met^ lacking m^1^A58 by the RTD pathway was recently shown to be conserved in the phylogenetically distant yeast species *S. pombe* and *S. cerevisiae* (42), suggesting that throughout eukaryotes the m^1^A58 modification is crucial to tRNA_i_^Met^ biology.

Here, aiming to reconcile the apparent inconsistency regarding the incorporation of m^1^A58 in yeast tRNAs, namely as a late modification in elongator tRNA^Phe^ and as an early modification in initiator tRNA_i_^Met^, we decided to examine the m^1^A58 modification pathways in yeast elongator and initiator tRNAs (see Supplementary Figure S1 for the sequence and modifications of yeast tRNA^Phe^ and tRNA_i_^Met^). On the elongator tRNA^Phe^, we aimed at characterizing the molecular details related to the modification circuits present in the T-loop and involving Ψ55, T54 and m^1^A58, in order, in particular, to untangle direct from indirect effects. On the initiator tRNA_i_^Met^, we sought to investigate the introduction of m^1^A58 and its dependence on other modifications. In addition, we aimed at investigating the impact of the m^1^A58 modification on the structural properties of the tRNA_i_^Met^ elbow region. Understanding the maturation process of initiator tRNA_i_^Met^, and in particular the m^1^A58 incorporation, which has consequences on its stability and quality control, is indeed crucial considering the central role of tRNA_i_^Met^ in translation initiation and hence gene expression.

For that, we first implemented a generic approach enabling the preparation of tRNAs containing specific modifications. We then used these specifically modified tRNAs to demonstrate that the incorporation of T54 in tRNA^Phe^ is directly stimulated by Ψ55, and that the incorporation of m^1^A58 in tRNA^Phe^ is directly and individually stimulated by Ψ55 and T54, with a remarkable cumulative effect when they are present together, thereby reporting in detail the molecular mechanisms controlling the Ψ55 $ \to$ T54 $ \to$ m^1^A58 modification circuit in yeast elongator tRNAs. We also show that m^1^A58 is efficiently introduced on unmodified tRNA_i_^Met^, and does not strictly need any prior modification, although m^5^C48,49 have a slight stimulatory effect on m^1^A58 incorporation. Finally, we show that the m^1^A58 single modification has major effects on the structural properties of yeast tRNA_i_^Met^, with the tRNA elbow structure being properly assembled only when this modification is present. This provides a structural basis to the degradation of hypomodified tRNA_i_^Met^ lacking m^1^A58 by the nuclear surveillance and RTD pathways.

## MATERIALS AND METHODS

### Yeast strains

Yeast strains used in this study are listed in Supplementary Table S1. The wild-type *S. cerevisiae* BY4741 strain and the YKO collection kanMX strains carrying deletions of the genes for modification enzymes Trm1, Trm2, Trm4, Trm8, Trm10, Trm11, Pus4, Dus1, Dus3 and Rit1, were obtained from Euroscarf and used for tRNA preparations for MS analysis. The proteinase-deficient *S. cerevisiae* strain c13-ABYS-86 and the derived strain c13-ABYS-86-*trm4Δ* were used for the preparation of yeast extracts for NMR experiments. All strain constructions were verified by PCR using appropriate oligonucleotides (listed in Supplementary Table S2).

### *E*. *coli* strains

*E. coli* strains used in this study are listed in Supplementary Table S1. The *E. coli* BL21(DE3) CodonPlus-RIL *yggH::kan* (trmB) strain was constructed by transferring the *yggH::kan* cassette from the appropriate K-12 strain of the Keio collection (44) to a BL21(DE3) CodonPlus-RIL strain (Agilent) by phage P1 *vir*-mediated transduction (45) (Supplementary Table S1). Deletion of the *yggH* gene and its replacement by the kanamycin resistance cassette in the BL21(DE3) CodonPlus-RIL strain was checked with PCR using appropriate sets of primers (Supplementary Table S2).

### Modification enzymes cloning

The gene encoding the full-length yeast Pus4 (M1 to V403 – Uniprot entry P48567) was cloned from BY4741 genomic DNA between the *EcoRI* and *NotI* sites of a modified pET28a vector (Novagen) encoding an N-terminal His_6_-tag cleavable with TEV protease (pET28-Pus4). The gene encoding the full-length yeast Trm2 (M1 to I639 – Uniprot entry P33753) was initially cloned from BY4741 genomic DNA between the *EcoRI* and *NotI* sites of a pGEX-6p-1 vector (pGEX-Trm2). However, this construct was insoluble and poorly expressed in *E. coli* BL21(DE3) CodonPlus-RIL cells. Since the N-terminal part of Trm2 contains highly hydrophobic stretches of amino-acids, and does not correspond to the catalytic domain of the protein, a second construct corresponding to V116 to I639 was cloned between the *BamHI* and *XhoI* sites of a pRSFDuet-Smt3 vector leading to an N-terminal His_6_-SUMO- fusion of Trm2 (pSUMO-Trm2). The naturally present *BamHI* site in the yeast *trm2* gene was first removed by a silent mutation of the codon encoding for D564 from GAT to GAC with site directed mutagenesis. The genes encoding yeast Trm6/Trm61 heterodimer (Trm6: M1 to I478 – Uniprot entry P41814; Trm61: M1 to K383 – Uniprot entry P46959) were cloned from BY4741 genomic DNA between the *BamHI* and *NotI* sites for Trm6 and *NdeI* and *XhoI* sites for Trm61 of a pETDuet-1 vector (Novagen) thereby encoding an N-terminal His_6_-tag on Trm6 (pETDuet-Trm6/Trm61).

### Modification enzymes purification

Pus4, Trm2 and Trm4 were overexpressed in *E. coli* BL21(DE3) CodonPlus-RIL cells (Agilent) in LB media. Trm6/Trm61 heterodimer was overexpressed in *E. coli* BL21(DE3) CodonPlus-RIL *yggh::kan* cells lacking the *E. coli* enzyme catalyzing m^7^G46 modifications in tRNAs, namely TrmB since initial expression and purification in *E. coli* BL21(DE3) CodonPlus-RIL cells lead to a Trm6/Trm61 heterodimer contaminated with an m^7^G46 modification activity (see Supplementary Figure S2). The cells were grown at 37°C to OD_600_ ∼0.4, cooled down to 18–30°C and induced at OD_600_ ∼0.6 by adding (IPTG) to a final concentration of 0.4–0.5 mM. Cells were harvested 6–22 h after induction by centrifugation. Cell pellets were resuspended in the corresponding lysis buffer supplemented with an EDTA-free antiprotease tablet (Roche) and lysed by sonication. Cell lysates were centrifuged for 30 min at 35 000 g. All column chromatography purifications were performed on a ÄKTA Pure purification system (Cytiva) at 4°C. The cell lysate supernatant was loaded on a Ni-NTA column and the protein of interest was eluted with an imidazole gradient. Fractions containing the protein were pooled, concentrated with an Amicon 50 000 MWCO (Millipore) and further purified with a combination of hydrophobic and size exclusion chromatography depending on the protein. Purified protein samples loaded on size exclusion chromatography were eluted in the corresponding protein storage buffer, confirmed for purity using SDS-PAGE (Supplementary Figure S3), concentrated with an Amicon (Millipore) to ∼5–10 mg/ml and stored at –20°C. The protein concentrations were determined by absorbance at 280 nm using the corresponding mM extinction coefficient (See Supplementary Table S3 for specific details on the purifications).

### RNA sample preparation for NMR and enzyme activity assays

Unmodified yeast tRNA^Phe^-WT, tRNA_i_^Met^-WT, tRNA^Phe^-ΔU17, tRNA^Phe^-A20A60, tRNA^Phe^-A54, tRNA^Phe^-UAAA, tRNA_i_^Met^-U17, tRNA_i_^Met^-G20C60, tRNA_i_^Met^-U54 and tRNA_i_^Met^-UGCU were prepared by standard *in vitro* transcription following previously published procedures, either with unlabelled NTPs or ^15^N-labelled Us and Gs (39,46). We replaced the first Watson Crick base pair A1-U72 of tRNA_i_^Met^ with a G1-C72 base pair in order to improve *in vitro* transcription efficiency. To prepare the single modified Ψ55-tRNA^Phe^, 112 μM of refolded tRNA^Phe^ was incubated with 3.3 μM of purified Pus4 for 40 min at 30°C in an 800 μl reaction mix. To prepare T54-tRNA^Phe^, 80 μM of refolded tRNA^Phe^ was incubated with 12 μM of purified Trm2 and a ∼6–8-times excess of S-adenosyl-L-methionine (SAM) in an 800 μl reaction mix for 14 h at 30°C. To prepare the double modified Ψ55-T54-tRNA^Phe^, 80 μM of Ψ55-tRNA^Phe^ was incubated with 8 μM Trm2 and a ∼6–8-times excess of SAM in an 800 μl reaction mix for 4 h at 30°C. To prepare m^5^C48,49-tRNA_i_^Met^, 146 μM of refolded unmodified-tRNA_i_^Met^ was incubated with 23 μM of purified Trm4 for 17 h at 30°C in a 500 μl reaction mix. All reactions were performed in the following maturation buffer (MB): 100 mM NaH_2_PO_4_/K_2_HPO_4_ pH 7.0, 5 mM NH_4_Cl, 2 mM DTT and 0.1 mM EDTA. The tRNA reaction products were then purified by ion exchange chromatography (MonoQ, Cytiva), dialyzed extensively against 1 mM Na-phosphate pH 6.5, and refolded by heating at 95°C for 5 min and cooling down slowly at room temperature. Buffer was added to place the tRNAs in the NMR buffer (10 mM Na-phosphate pH 6.5, 10 mM MgCl_2_), and the samples were concentrated using Amicon 10000 MWCO (Millipore) to ∼80 μM for further use in kinetic assays, or ∼1.4–1.5 mM for the NMR study of tRNA_i_^Met^ maturation in yeast extracts.

### Trm2 and Trm6/Trm61 kinetic assays on different substrates

To measure initial velocities of m^1^A58 and T54 formation, 10 μM of unmodified tRNA^Phe^, Ψ55-tRNA^Phe^, T54-tRNA^Phe^, Ψ55-T54-tRNA^Phe^, unmodified tRNA_i_^Met^ and m^5^C48,49-tRNA_i_^Met^ were incubated each in a 300 μl reaction with enzyme concentrations varying from 50 to 300 nM depending on enzyme and substrate type, 18 μM non-radioactive SAM and 50 nM of radioactive [^3^H]-SAM (see Supplementary Table S4 for details on the reaction mixes). Reactions were performed in the MB buffer and were incubated at 30°C for 30 min except for the reaction with Trm6/Trm61 and the unmodified tRNA^Phe^ that was incubated for 96 min. Aliquots of 50 μl were taken of each reaction at 6, 12, 18, 24 and 30 min (for the 30 min reactions) and at 24, 48, 72 and 96 min (for the 96 min reaction) and the samples were quenched by adding 5% (v/v) cold trichloracetic acid (TCA). Quenched samples were filtered through Whatman glass microfibers disks pre-soaked with 5% (v/v) TCA, washed four times with 5% (v/v) TCA and one final time with ethanol. The filter disks were dried, then 5 ml Optiphase ‘HISAFE’ 2 scintillation cocktail (PerkinElmer) were added, and the counts per minute (CPM) equivalent to the incorporated [^3^H]-methyl were determined by scintillation counting. Then CPM values were converted to concentrations of modified tRNAs using [^3^H]-SAM/CPM calibration standards. Enzymatic reactions were performed in triplicates or quadruplicates. Since Trm6/Trm61 and Trm2 activity turned out to vary greatly between different substrates, different enzyme concentrations were used to perform the kinetic assays. Therefore, we normalized the quantities of modified tRNAs to an equivalent of 50 nM of enzyme. Initial velocities (V_i_) were determined by linear regression using Prism7 (GraphPad), i.e. data were fitted to a single linear function: *y* = *V_i_* .*x* while forcing the curve to pass through the origin, and standard errors (SE) on the *V_i_* were determined by taking into account the data spread.

### Trm6/Trm61 activity assays on yeast tRNA^Phe^ and tRNA_i_^Met^ variants

To measure m^1^A58 formation, 10 μM of unmodified yeast tRNA^Phe^-WT, tRNA^Phe^-ΔU17, tRNA^Phe^-A20A60, tRNA^Phe^-A54, tRNA^Phe^-UAAA, tRNA_i_^Met^-WT, tRNA_i_^Met^-U17, tRNA_i_^Met^-G20C60, tRNA_i_^Met^-U54 and tRNA_i_^Met^-UGCU were incubated each in a 100 μl reaction with 600 nM of purified Trm6/Trm61, 18 μM non-radioactive SAM and 100 nM of radioactive [^3^H]-SAM. Reactions were performed in the MB buffer at 30°C and concentrations of modified tRNAs were measured at *t* = 60 min. Samples were then treated as described above. Enzymatic reactions were performed in six replicates (*N* = 6). Standard deviations were relatively uniform across the different tRNA substrates and corresponded to 20–33% of the average value for tRNA^Phe^ variants and to 17–20% for tRNA_i_^Met^ variants. CPM values were converted to concentrations of modified tRNAs using [^3^H]-SAM/CPM calibration standards.

### NMR spectroscopy

All NMR spectra of yeast tRNA^Phe^ and tRNA_i_^Met^ were measured at 38°C on a Bruker AVIII-HD 700 MHz spectrometer equipped with TCI 5-mm cryoprobe with 5-mm Shigemi tubes in the NMR buffer (10 mM Na-phosphate pH 6.5, 10 mM MgCl_2_) supplemented with 5% (v/v) D_2_O. To verify that the desired modifications were incorporated quantitatively in yeast tRNA^Phe^, 1D jump-and-return-echo NMR spectra (47,48) of the different tRNAs were measured and compared to previously characterized samples (39,49). To analyse the effect of nucleotide swapping on the structural properties of yeast tRNA^Phe^ and yeast tRNA_i_^Met^, 2D (^1^H,^15^N)-BEST-TROSY spectra of unmodified yeast tRNA^Phe^-WT, tRNA^Phe^-ΔU17, tRNA^Phe^-A20A60, tRNA^Phe^-A54, tRNA^Phe^-UAAA, tRNA_i_^Met^-WT, tRNA_i_^Met^-U17, tRNA_i_^Met^-G20C60, tRNA_i_^Met^-U54 and tRNA_i_^Met^-UGCU were measured at 38°C in the NMR buffer. In addition, to evaluate the effect of specific modifications on the structural properties of yeast tRNA_i_^Met^, 2D (^1^H,^15^N)-BEST-TROSY spectra of unmodified tRNA_i_^Met^, m^5^C48,49-tRNA_i_^Met^ and m^1^A58-tRNA_i_^Met^ were measured at 38°C in the NMR buffer. Imino resonances of the m^1^A58-tRNA_i_^Met^ were assigned using 2D jump-and-return-echo (^1^H,^1^H)-NOESY (47,48) and 2D (^1^H,^15^N)-BEST-TROSY (50) experiments. For monitoring the maturation of tRNA_i_^Met^ in yeast extract, wild-type and *trm4Δ* yeast extracts were prepared in the c13-ABYS-86 background, as previously described (40). NMR spectra were measured at 30°C with unmodified ^15^N-[U/G]-labelled tRNA_i_^Met^ at 40 μM in yeast extracts supplemented with NaH_2_PO_4_/K_2_HPO_4_ pH 6.5 150 mM, NH_4_Cl 5 mM, MgCl_2_ 5 mM, DTT 2 mM, EDTA 0.1 mM, SAM 4 mM, ATP 4 mM, NADPH 4 mM and D_2_O 5% (v/v) (51). Each 2D (^1^H,^15^N)-BEST-TROSY experiment of the series was measured with a recycling delay of 200 ms, a SW(^15^N) of 26 ppm and 96 increments for a total experimental time of 120 min. The data were processed using TOPSPIN 3.6 (Bruker) and analysed with Sparky (http://www.cgl.ucsf.edu/home/sparky/).

### Total tRNA samples from yeast for mass spectrometry

Total tRNA from *S. cerevisiae* BY4741 wild-type or mutant strains used for mass spectrometry analysis were prepared as described previously (39). For each strain, all cultures and tRNA preparations were performed in triplicate for statistical analysis. Yeast tRNA_i_^Met^ was isolated from ∼1 μg total tRNA samples with a first step of SEC and a subsequent purification using T1 Dynabeads (Thermo Fisher Scientific, Product no. 65801D) and a DNA probe specific to tRNA_i_^Met^ ([Btn]- AAA-TCG-GTT-TCG-ATC-CGA-GGA-CAT-CAG-GGT-TAT-GA, Sigma-Aldrich, Munich, Germany) as previously reported (39,52,53).

### Digestion of tRNAs to nucleosides and quantification by mass spectrometry

Purified tRNA_i_^Met^ samples were digested to single nucleosides following previously published procedures (39) and stable isotope-labelled internal standard (SILIS, 0.1 volume of 10X solution) from yeast was added for absolute quantification (54). Quantification of the m^1^A modification in tRNA_i_^Met^ was performed with an Agilent 1290 Infinity II equipped with a DAD combined with an Agilent Technologies G6470A Triple Quad system and electro-spray ionization (ESI-MS, Agilent Jetstream) following previously published procedures (39,54). Absolute abundance of m^1^A from wild-type yeast corresponded to 0.69 ± 0.04 m^1^A per tRNA_i_^Met^. The absolute quantities of m^1^A in the deleted strains were normalized to that of the wild-type strain to determine abundance relative to wild-type. Analyses of the variations compared to the wild-type strain were conducted from the determination of the confidence intervals at 95% (CI 95%) using Prism7 (GraphPad).

## RESULTS

### A generic approach to prepare tRNAs with specific modifications

**Figure 1.**
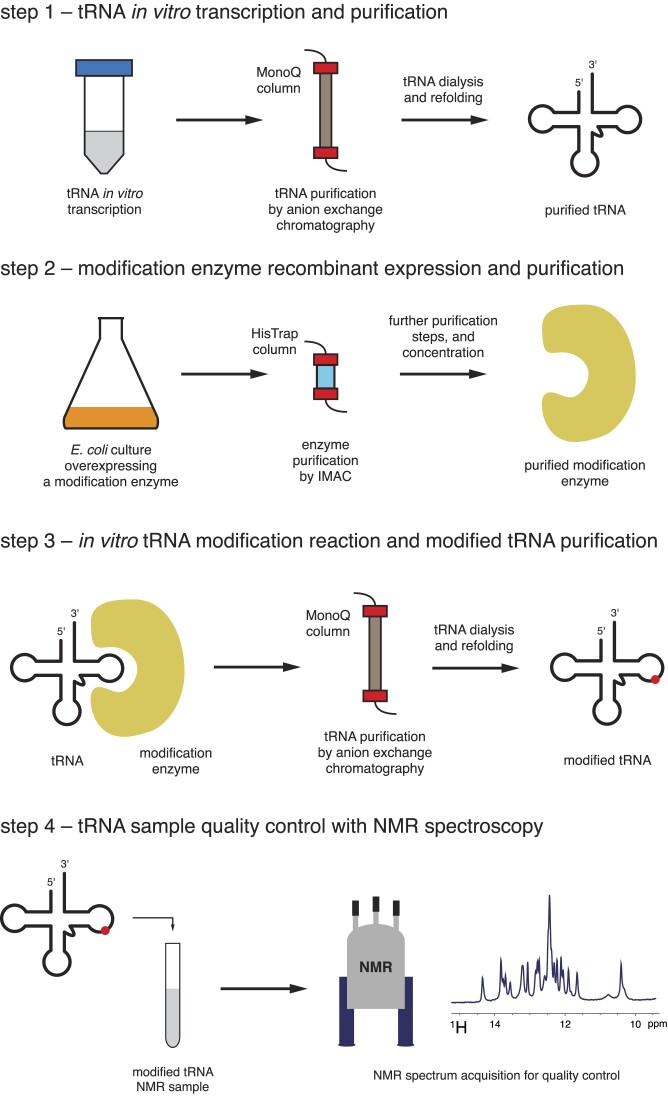
A generic approach to prepare tRNAs with specific modifications. (Step 1) The unmodified tRNA is transcribed *in vitro* and purified by anion exchange chromatography. (Step 2) The desired modification enzyme is overexpressed in *E. coli* and purified by immobilized metal affinity chromatography (IMAC) and further purification steps if needed. (Step 3) The unmodified tRNA is modified *in vitro* with the purified modification enzyme in presence of cofactors and subsequently purified by anion exchange chromatography. (Step 4) A quality control step is performed by 1D ^1^H NMR in order to establish that the desired modifications were fully incorporated in the tRNA population.

In order to evaluate the effect of pre-existing modifications on the introduction of further ones, we have implemented a generic method for preparing tRNA samples with a single or a specific set of modifications (Figure 1). Our approach is divided into four successive steps, (1) tRNA *in vitro* transcription and purification, (2) modification enzyme expression and purification, (3) *in vitro* tRNA modification reaction and modified tRNA purification and (4) tRNA sample quality control by NMR spectroscopy (Figure 1). To introduce several modifications on a tRNA, steps 3 and 4 can be reiterated on a tRNA sample already carrying modification(s).

For the present study on yeast tRNA^Phe^ and tRNA_i_^Met^, in addition to the unmodified tRNA^Phe^ and tRNA_i_^Met^, we applied our methodology to produce: tRNA^Phe^ samples carrying single modifications (Ψ55-tRNA^Phe^ and T54-tRNA^Phe^), or double modifications (Ψ55-T54-tRNA^Phe^), and tRNA_i_^Met^ samples carrying m^5^C48,49 or m^1^A58 modifications (m^5^C48,49-tRNA_i_^Met^ and m^1^A58-tRNA_i_^Met^). For this purpose, we first transcribed and purified, using anion exchange chromatography, the yeast unmodified tRNA^Phe^ and tRNA_i_^Met^ (Figure 1, step 1). We then overexpressed and purified the yeast enzymes Pus4 that introduces Ψ55, Trm2 that adds T54 (or m^5^U54), Trm6/Trm61 that adds m^1^A58 and Trm4 that introduces m^5^C48 and m^5^C49 (see Materials and Methods and Supplementary Figure S1; Figure 1, step 2). Next, preliminary activity tests with these different enzymes allowed us to estimate the enzyme to tRNA ratios and the incubation times needed to introduce the desired modifications quantitatively. We thus incubated the unmodified tRNAs with the appropriate enzymes and cofactors for the required duration, and then purified the *in vitro* modified tRNAs using anion exchange chromatography (Figure 1, step 3). Finally, we verified that the desired modifications were introduced quantitatively by performing a quality control of our samples with NMR spectroscopy (Figure 1, step 4).

### The introduction of T54 by Trm2 to the yeast tRNA^Phe^ is stimulated by Ψ55

**Figure 2.**
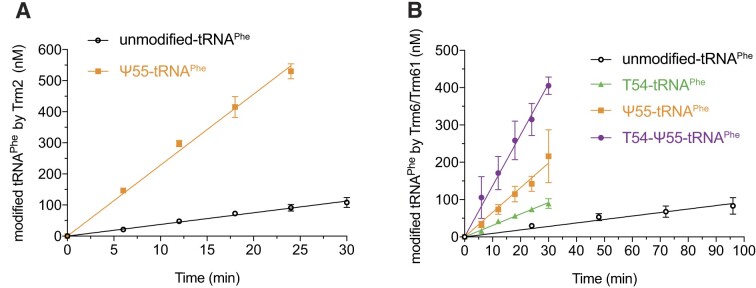
Influence of pre-existing modifications on Trm2 and Trm6/Trm61 activities on tRNA^Phe^. (**A**) Time course of the introduction of T54 in tRNA^Phe^ depending on the prior presence (orange) or absence (black) of the Ψ55 modification. (**B**) Time course of the introduction of m^1^A58 in tRNA^Phe^ depending on pre-existing modifications: unmodified tRNA^Phe^ (black), single modified T54-tRNA^Phe^ (green) and Ψ55-tRNA^Phe^ (orange), and double modified T54-Ψ55-tRNA^Phe^ (purple). Modified tRNA quantities were measured for 4 or 5 time points in at least three independent experiments (*N* = 3 or 4), and initial velocities (*V_i_*) were determined by linear regression (see Tables 1 and 2).

The fact that we observed a slower incorporation of T54 in tRNA^Phe^ in the *pus4Δ* yeast extract, and that the amount of T54 in tRNA^Phe^ as well as in the total tRNA population is drastically reduced in the *pus4Δ* strain, suggested that the Ψ55 modification had a positive effect on the introduction of T54 by Trm2 (39). However, we could not exclude that the defect in T54 incorporation was due to a negative effect of other modification(s) that only become apparent in the absence of Ψ55 or that the genetic expression of Trm2 was affected in the *pus4Δ* strain. Here, in order to unambiguously determine whether the introduction of T54 on the yeast tRNA^Phe^ by Trm2 is directly dependent on the presence of Ψ55, we conducted activity assays with Trm2 on unmodified tRNA^Phe^ and Ψ55-tRNA^Phe^ (produced as described above). Trm2 was incubated with each of the tRNAs in the presence of the methyl-donor cofactor SAM carrying a radioactive methyl group (*S*-adenosyl-l-methionine [methyl-^3^H]), and aliquots were taken at different time points to determine the initial velocities (*V_i_*) of the methylation reactions (Figure 2A, Table 1, and Supplementary Figure S4). These activity assays clearly demonstrated that the methylation reaction catalysed by Trm2 is about 6 times faster on the Ψ55-tRNA^Phe^ when compared to the unmodified tRNA^Phe^ (Table 2). This shows that the catalytic efficiency of Trm2 introducing T54 to tRNA^Phe^ directly depends on the prior presence of Ψ55 and establishes the direct positive link between Ψ55 and the introduction of T54 by Trm2.

**Table 1.** Initial velocities (*V_i_*) of Trm2 and Trm6/Trm61 acting on yeast tRNAs presenting different modification profiles. Initial velocities were determined by linear regression and normalized to an equivalent of 50 nM of enzyme. The reported errors correspond to the standard error (SE) of the slope determination (see material and methods)

Enzyme	Trm2		Trm6/Trm61					
Yeast tRNAs	tRNA^Phe^	Ψ55-tRNA^Phe^	tRNA^Phe^	T54-tRNA^Phe^	Ψ55-tRNA^Phe^	Ψ55-T54-tRNA^Phe^	tRNA_i_^Met^	m^5^C-tRNA_i_^Met^
*V_i_* (nM/min)	3.8 ± 0.1	22.9 ± 0.5	0.93 ± 0.05	3.1 ± 0.1	6.6 ± 0.3	13.7 ± 0.3	10.7 ± 0.3	15.2 ± 0.4

**Table 2.** Ratios of initial velocities (*V_i_*) showing enzyme efficiency depending on the presence of pre-existing modifications on the yeast tRNA^Phe^ and tRNA_i_^Met^. The reported errors of the ratios were calculated by taking into account the propagation of uncertainties

Enzyme	Trm2	Trm6/Trm61						
Yeast tRNAs	Ψ55-tRNA^Phe^/ tRNA^Phe^	T54-tRNA^Phe^/ tRNA^Phe^	Ψ55-tRNA^Phe^/ tRNA^Phe^	Ψ55-T54-tRNA^Phe^/ tRNA^Phe^	m^5^C-tRNA_i_^Met^/ tRNA_i_^Met^	tRNA_i_^Met^/ tRNA^Phe^	tRNA_i_^Met^/ Ψ55-T54-tRNA^Phe^	m^5^C-tRNA_i_^Met^/ Ψ55-T54-tRNA^Phe^
Vi ratio	6.1 ± 0.2	3.3 ± 0.2	7.1 ± 0.5	15 ± 0.8	1.4 ± 0.1	11.5 ± 0.7	0.8 ± 0.03	1.1 ± 0.04

### The introduction of m^1^A58 by Trm6/Trm61 to the yeast tRNA^Phe^ is stimulated by Ψ55 and T54

Likewise, our previous work suggested a positive effect of the Ψ55 and T54 modifications on the introduction of m^1^A58 by the Trm6/Trm61 complex (39). However, as explained above for Trm2, we could not exclude that the observed behaviours were due to alternative effects. In addition, considering the above-mentioned effect of Ψ55 on T54, it was not possible to distinguish a direct effect of Ψ55 on m^1^A58 from an indirect effect via T54. To definitely establish whether the introduction of m^1^A58 on the yeast tRNA^Phe^ is directly dependent on the presence of Ψ55 and T54, we conducted activity assays with the Trm6/Trm61 complex on unmodified tRNA^Phe^, Ψ55-tRNA^Phe^, T54-tRNA^Phe^ and Ψ55-T54-tRNA^Phe^. The Trm6/Trm61 complex was incubated with each of the tRNAs in the presence of a radioactive [methyl-^3^H]-SAM cofactor, and aliquots were taken at different time points to derive the initial velocities (Figure 2B, Table 1, and Supplementary Figure S5a–d). With these activity assays, we observed that the introduction of m^1^A58 by Trm6/Trm61 is 3.3 times more efficient when the T54 modification is present compared to the unmodified tRNA^Phe^, 7.1 times more efficient in the presence of Ψ55 and 15 times more efficient if both T54 and Ψ55 are present in the yeast tRNA^Phe^ (Table 2). This demonstrates that T54 and Ψ55 have individually a positive effect on the introduction of m^1^A58, as well as a cumulative positive effect if they are both simultaneously present. Therefore, the catalytic activity of Trm6/Trm61 directly depends on the presence of both the T54 and Ψ55 modifications. Additionally, our measurements indicate that Ψ55 stimulates the introduction of m^1^A58 about two times more efficiently than T54.

### The m^1^A58 modification is efficiently introduced on an unmodified tRNA_i_^Met^

The results presented above showed that efficient introduction of m^1^A58 in tRNA^Phe^, strongly depends on the prior presence of Ψ55 and T54 in the T-loop. In addition, since the levels of modifications observed for total yeast tRNAs and tRNA^Phe^, are similarly affected in the *pus4Δ* and *trm2Δ* strains, the stimulation effect of Ψ55 and T54 on the introduction of m^1^A58 is certainly a common feature of several yeast tRNAs (39). At first sight, it might seem paradoxical that the efficiency of an enzyme encoded by two essential genes, i.e. *trm6/trm61* (5,55,56), is highly dependent on the prior presence of modifications encoded by non-essential genes, i.e. *pus4* and *trm2*. However, the origin of the essentiality of the m^1^A58 modification has been studied in detail in yeast and has been shown to be related to its importance for the maturation of initiator tRNA_i_^Met^ (55). Hypomodified initiator tRNA_i_^Met^ lacking m^1^A58 are indeed targeted to degradation by RNA decay pathways (27,28,42). As a possible explanation to this paradox, we noted that yeast tRNA_i_^Met^ does not carry T54 and Ψ55 in the T-loop, but contains unmodified A54 and U55 (Supplementary Figure S1). Altogether, we anticipated that the initiator tRNA_i_^Met^ would have its own pathway of modification in the T-arm, in which the m^1^A58 modification did not depend on pre-existing modifications. More generally, since tRNA_i_^Met^ transcripts lacking m^1^A58 are degraded by RNA decay pathways, it seems reasonable that levels of m^1^A58 in tRNA_i_^Met^ should not be altered in different strains or growth conditions. Modification levels should indeed reflect the requirement of m^1^A58 for tRNA_i_^Met^ stability.

**Figure 3.**
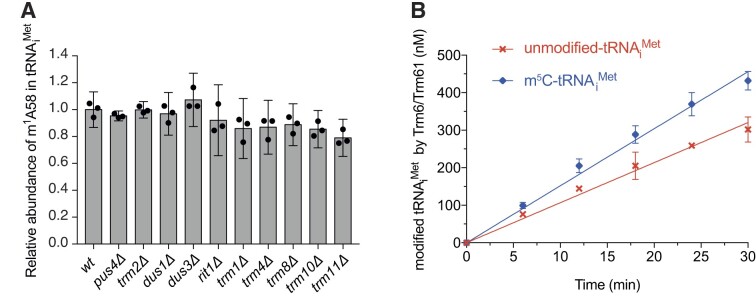
Influence of pre-existing modifications on m^1^A58 abundance and on Trm6/Trm61 activity on tRNA_i_^Met^. (**A**) Quantitative analysis of nucleoside modifications in yeast tRNA_i_^Met^ with LC–MS/MS. Histograms showing the relative abundance of m^1^A58 modification in purified yeast tRNA_i_^Met^ prepared from modification-enzyme-deleted strains using the wild-type levels as reference. Black dots represent individual measurements, data heights represent the mean of the biological replicates. Error bars correspond to the confidence interval at 95% (CI 95%). Modifications were quantified in three independent biological replicates (*N* = 3). (**B**) Time course of the introduction of m^1^A58 in tRNA_i_^Met^ depending on the prior presence (blue) or absence (red) of the m^5^C48,49 modifications. Modified tRNA quantities were measured for 5 time points in three independent experiments (*N* = 3), and initial velocities (*V_i_*) were determined by linear regression (see Tables 1 and 2).

To examine these points, we measured, using LC–MS/MS, the levels of m^1^A in tRNA_i_^Met^ from *pus4Δ* and *trm2Δ* strains, and from *dus1Δ*, *dus3Δ*, *rit1Δ*, *trm1Δ*, *trm4Δ*, *trm8Δ*, *trm10Δ* and *trm11Δ* strains, involved in the introduction of modifications D16, D47, Ar(p)64, m^2^_2_G26, m^5^C48,49, m^7^G46, m^1^G9 and m^2^G10, respectively. These levels were compared with the levels of m^1^A in tRNA_i_^Met^ from wild-type yeast cultured under the same experimental conditions. As expected, we observed no substantial changes in the amount of m^1^A in any of these deleted strains compared with the wild-type level (Figure 3A). The slight variations observed between some deleted strains and the wild-type could reflect a certain stability of m^1^A58-depleted tRNA_i_^Met^ and/or small variations in the degree of purity of tRNA_i_^Met^ recovered from the total tRNA population in the purification procedure. In any case, our data do not allow to conclude that these slight variations are significant. Overall, this shows that the lack of any other single modification does not prevent the formation of mature tRNA_i_^Met^ carrying m^1^A58, and suggests that m^1^A58 can be correctly introduced on unmodified tRNA_i_^Met^.

To evaluate the efficiency of m^1^A58 modification on an unmodified tRNA_i_^Met^, we conducted activity assays with Trm6/Trm61 on unmodified tRNA_i_^Met^ produced by *in vitro* transcription as described for tRNA^Phe^ (Figure 3B, Table 1, and Supplementary Figure S5e). We observed that the introduction of m^1^A58 by Trm6/Trm61 is 11.5 times more efficient on the unmodified tRNA_i_^Met^ than on the unmodified tRNA^Phe^ (Table 2). This rate corresponds to an efficiency of about 0.8 times that measured on the doubly-modified Ψ55-T54-tRNA^Phe^ (Table 2). Our data therefore establish that, on the contrary to its introduction on unmodified tRNA^Phe^, m^1^A58 is efficiently introduced on unmodified tRNA_i_^Met^, with an efficiency that is comparable to that observed for an optimally modified tRNA^Phe^ bearing both Ψ55 and T54.

### A54 is required for an efficient incorporation of m^1^A58 on unmodified tRNA_i_^Met^

Aiming to identify the sequence elements and associated structural properties implicated in the differences observed for m^1^A58 incorporation in tRNA^Phe^ and tRNA_i_^Met^, we designed a set of tRNA variants with the objective of transfering elongator sequence elements and associated structural properties to initiator tRNA_i_^Met^, and vice versa. Since m^1^A58 is part of the specific initiator elbow structure (43), residues involved in this unique substructure, namely A20, A54 and A60, were primarily targeted for mutations. In addition, since the absence of nucleotide U17 is also a characteristic of initiator tRNA_i_^Met^, we chose to add it in a tRNA_i_^Met^ variant. All tRNA variants, with their specific mutation or set of mutations are schematically summarized on Supplementary Figure S6.

First, in order to evaluate the effect of the nucleotide swapping between tRNA^Phe^ and tRNA_i_^Met^ from a structural point of view, we conducted NMR analysis on each tRNA variant. The comparison of the NMR fingerprint of unmodified tRNA^Phe^ and tRNA_i_^Met^ revealed clear differences (Supplementary Figure S6). The NMR spectrum of tRNA^Phe^-WT displays sharp and uniform NMR signals, characteristic of a stable, homogeneously folded tRNA. On the contrary, the NMR spectrum of tRNA_i_^Met^-WT exhibits a heterogeneous NMR signal profile with both weak and strong signals, as well as signals with atypic line shapes. These classic exchange-broaden signals reflect a less homogeneous folding for unmodified tRNA_i_^Met^ that probably exchanges between several folding states, an exchange occurring in the intermediate regime relative to the NMR chemical shift time scale. The NMR fingerprints of the different variants revealed that for tRNA^Phe^, important structural changes are taking place in the tRNA^Phe^-A54 and tRNA^Phe^-UAAA variants, which tend to acquire a heterogeneous NMR spectrum profile (Supplementary Figure S6a). Conversely, for tRNA_i_^Met^, structural changes are apparent mostly for the tRNA_i_^Met^-U54 and tRNA_i_^Met^-UGCU variants, which exhibit slightly less heterogeneous or atypic NMR line shapes (Supplementary Figure S6b).

**Figure 4.**
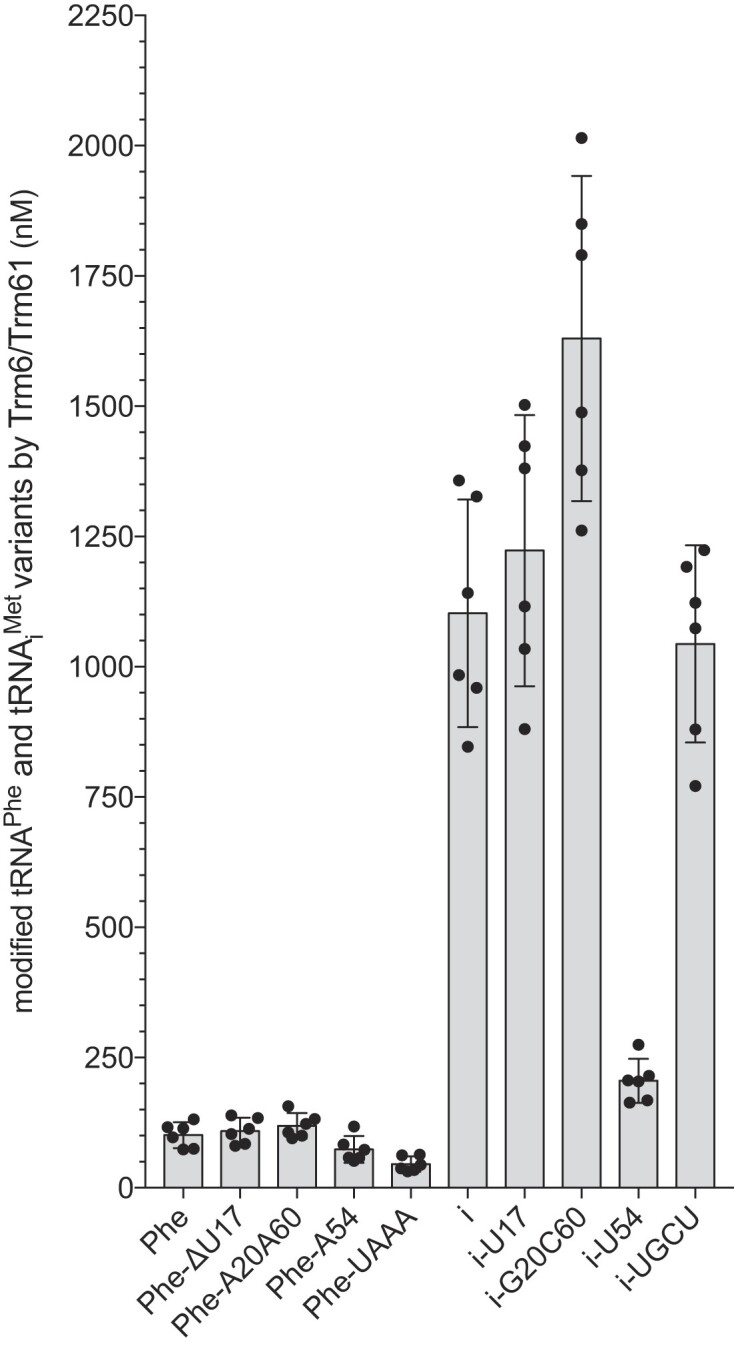
Influence of specific nucleotide swapping between tRNA^Phe^ and tRNA_i_^Met^ on Trm6/Trm61 activity. Histogram comparing the quantity of m^1^A58 introduced by Trm6/Trm61 in tRNA^Phe^ and tRNA_i_^Met^ variants (in nM). Names of the tRNAs are indicated below the graph and correspond to a specific nomenclature (see Supplementary Figure S6 for correspondence and details). Black dots represent individual measurements. Modified tRNA quantities were measured for 1 time point at *t* = 1 h in six independent experiments (*N* = 6). Data heights represent the mean of the replicates. Error bars correspond to the confidence interval at 95% (CI 95%).

Next, we conducted activity assays with Trm6/Trm61 on unmodified tRNA^Phe^ and tRNA_i_^Met^ variants. On one hand, we observed that Trm6/Trm61 is ∼5.5 times less efficient on tRNA_i_^Met^-U54, where A54 is replaced by U54, compared to tRNA_i_^Met^-WT (Figure 4). This shows that A54 is required for an efficient incorporation of m^1^A58 by Trm6/Trm61 on unmodified tRNA_i_^Met^. We do not observe any other significant changes in the efficiency of m^1^A58 introduction on other tRNA_i_^Met^ variants and in particular on tRNA_i_^Met^-UGCU (Figure 4). This is quite puzzling since tRNA_i_^Met^-UGCU also lacks the A54 residue. The additional mutations U17, G20 and C60 seem to neutralize the negative effect of the lack of A54. This demonstrates the inherent complexity and challenge associated with comprehending how nucleotides collaborate to establish intricate networks of interactions that shape the structure of tRNAs. On the other hand, the tRNA^Phe^-A54 variant, in which U54 is replaced by A54, does not show an increased efficiency of m^1^A58 incorporation (Figure 4). This is a good illustration that converting a good substrate into a poor substrate through the removal of a single key element is considerably simpler compared to the transformation of a poor substrate into a good one by introducing the same key element. In addition, we do not observe any changes in the efficiency of m^1^A58 introduction on tRNA^Phe^-ΔU17 and tRNA^Phe^-A20A60 variants compared to tRNA^Phe^-WT. This shows that neither adding nor removing U17, A20 and A60 residues to either tRNA_i_^Met^ or tRNA^Phe^ has any effect on m^1^A58 formation. Although determining the sequence and structural elements that govern Trm6/Trm61 activity appeared complicated, we identified nucleotide A54, which interacts with the Hoogsteen face of the target A58, as a key element for the efficient introduction of m^1^A58 into tRNA_i_^Met^.

### The introduction of m^1^A58 by Trm6/Trm61 to the yeast tRNA_i_^Met^ is slightly stimulated by m^5^C48,49

After studying the structural effects on the introduction of m^1^A58 by mutating nucleotides implicated in the unique tRNA_i_^Met^ elbow structure, we wondered whether more subtle alterations that may also affect the tRNA_i_^Met^ local structure could modulate m^1^A58 incorporation. In particular, posttranscriptional modifications that are close in space to m^1^A58, and that participate in the tRNA_i_^Met^ tertiary interactions, could be considered as prime targets. Among tRNA_i_^Met^ modifications, m^5^C48 meets these criteria. Indeed, m^5^C48 is relatively close to m^1^A58 in the tRNA_i_^Met^ structure (<10 Å), and m^5^C48 and m^1^A58 are together implicated in the particular tRNA elbow structure of tRNA_i_^Met^ involving the previously mentioned non-canonical nucleotides A20, A54 and A60 (43). More precisely, m^5^C48 is involved in an intricate network of interactions with G15, A20 and A59, with A59 and A20 forming a relay with another network involving A60 and m^1^A58 (Supplementary Figure S7). Another aspect prompted us to examine the link between m^5^C modifications and m^1^A58. Indeed, since the yeast *trm4Δ* mutant has been implicated in the RTD pathway in combinations with several other mutations (24–26), and since hypomodified tRNA_i_^Met^ is targeted by the nuclear surveillance pathway and the RTD pathway, we wondered whether the modifications introduced by Trm4 could have an impact on the introduction of m^1^A58 by Trm6/Trm61, thereby affecting tRNA_i_^Met^ stability.

For these reasons, we investigated whether m^5^Cs have any effect on the introduction of m^1^A58 in tRNA_i_^Met^. Note that in yeast initiator tRNA_i_^Met^, Trm4 introduces m^5^Cs at two positions, namely m^5^C48 and m^5^C49 (Supplementary Figure S1). We therefore conducted activity assays with Trm6/Trm61 on m^5^C48,49-tRNA_i_^Met^. We observed that Trm6/Trm61 is about 40% more efficient in the presence of m^5^C48,49 as compared with the unmodified tRNA_i_^Met^ (Figure 3B, Table 1, and Supplementary Figure S5f). This corresponds to an efficiency of about 1.1 times the one measured on the doubly-modified Ψ55-T54-tRNA^Phe^ (Table 2). Thus, even though the m^5^C48,49 modifications are not strictly required for m^1^A58 introduction by Trm6/Trm61, their presence enhances the efficiency of m^1^A58 introduction in tRNA_i_^Met^*in vitro*.

**Figure 5.**
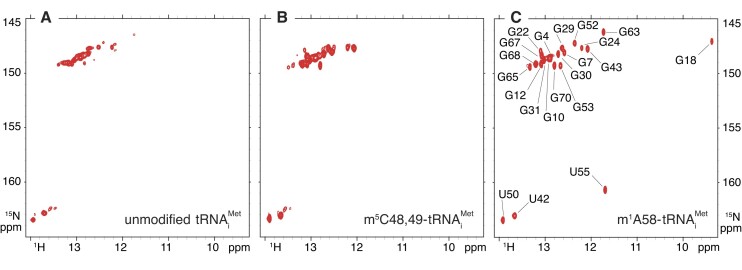
Effect of m^5^C48,49 and m^1^A58 on the structural properties of yeast tRNA_i_^Met^imino (^1^H,^15^N) correlation spectra of ^15^N-labelled tRNA_i_^Met^ with different modification status measured at 38°C. (**A**) unmodified tRNA_i_^Met^, **(B)** m^5^C48,49-tRNA_i_^Met^ and (**C**) m^1^A58-tRNA_i_^Met^. The assignment of the imino resonances of the m^1^A58-tRNA_i_^Met^ was obtained following standard methods.

In order to get a clearer idea of the origin of m^5^Cs positive effects on m^1^A58 introduction, we analysed m^5^C-containing tRNA_i_^Met^ with NMR spectroscopy. We produced an m^5^C48,49-tRNA_i_^Met^ sample ^15^N-labelled on its imino groups, thereby allowing for the measurements of 2D ^1^H–^15^N NMR spectrum, which corresponds to its NMR-fingerprint and reflects folding homogeneity and structural integrity, as explained previously for the tRNA^Phe^ and tRNA_i_^Met^ variants. The comparison of the ^1^H–^15^N BEST-TROSY experiments of unmodified and m^5^C48,49-tRNA_i_^Met^ samples revealed marked differences (Figure 5A, B). Additional signals appear on the spectrum of the m^5^C48,49-tRNA_i_^Met^, and a decrease in signal line broadening is observed. In addition, the signal heterogeneity present in the unmodified tRNA_i_^Met^, with weak and strong signals coexisting, is less pronounced in the m^5^C48,49-tRNA_i_^Met^ spectrum, which shows a more homogeneous signal profile, with overall stronger signals than in the unmodified tRNA_i_^Met^ (Figure 5A, B). As previously explained, NMR signals of RNA imino groups are only observed on condition that the imino protons are protected from exchange with the solvent by hydrogen bonding in any type of base pairing. The decrease in signal heterogeneity in the m^5^C48,49-tRNA_i_^Met^ therefore reflects more stable base pairs, as well as a less dynamic and more homogeneous folding of this tRNA. The introduction of m^5^Cs by Trm4 therefore induces local and/or global changes in the folding of tRNA_i_^Met^, which could explain the increased efficiency of m^1^A58 incorporation (Figure 3B, Tables 1 and 2).

### Effect of m^1^A58 on the structural properties of yeast tRNA_i_^Met^

Since m^1^A58 is involved in the particular tRNA elbow structure of tRNA_i_^Met^ (see (43) and text above), and since the m^1^A58 modification is essential for tRNA_i_^Met^ stability and prevents its degradation by the nuclear surveillance and RTD pathways (27,28,42), we examined the effect of this single modification on the structural properties of tRNA_i_^Met^. We thus produced a ^15^N-labelled m^1^A58-containing tRNA_i_^Met^ sample following our generic approach, and measured its NMR-fingerprint (Figure 5C). The comparison of the ^1^H-^15^N BEST-TROSY spectra of the unmodified and of the m^1^A58-tRNA_i_^Met^ (Figure 5A–C) revealed considerable changes in the structural properties of tRNA_i_^Met^ upon m^1^A58 modification. The pronounced signal heterogeneity present in unmodified tRNA_i_^Met^ (Figure 5A) is completely absent in m^1^A58-tRNA_i_^Met^ (Figure 5C), the NMR spectra of which display the characteristics of a stable and homogeneously folded tRNA. Thus, a single modification has major effects on the structural properties of yeast tRNA_i_^Met^, which can likely explain why hypomodified tRNA_i_^Met^ lacking m^1^A58 is targeted by degradation pathways.

To get a deeper understanding of the structural changes arising upon m^1^A58 introduction, we performed the assignment of the imino resonances of the m^1^A58-tRNA_i_^Met^ following standard methods (Figure 5C), as previously described for other tRNAs (49). With this assignment at hand, we noticed that the imino signals of G18 and U55 are only visible in the spectrum of the m^1^A58-tRNA_i_^Met^ (Figure 5A–C). These nucleotides, and their respective imino groups, are engaged in universally conserved tertiary interactions at the level of the elbow region of tRNAs, with the imino group of U55 forming a hydrogen bond with a non-bridging oxygen of the phosphate backbone of A58, and that of G18 forming a hydrogen bond with an exocyclic carbonyl group of U55 (57). The detection of these imino groups in the NMR spectra of m^1^A58-tRNA_i_^Met^ attests that their imino protons are protected from an exchange with the solvent, thereby demonstrating that the tRNA elbow structure is well-assembled. The imino signals of G18 and U55 can be considered as a signature of a properly folded tRNA with a well-assembled elbow structure. Conversely, their absence in the NMR-fingerprint of the unmodified tRNA_i_^Met^ and the m^5^C48,49-tRNA_i_^Met^ (Figure 5A, B) indicate that the tRNA elbow structure is not properly assembled in these tRNAs.

### Time-resolved NMR monitoring of m^1^A58 introduction in tRNA_i_^Met^ in yeast extract

**Figure 6.**
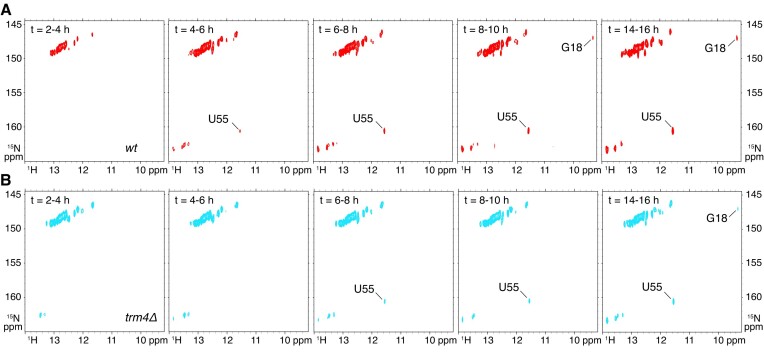
Time-resolved NMR monitoring of m^1^A58 introduction in tRNA_i_^Met^ in yeast extracts. (**A**) Imino (^1^H,^15^N) correlation spectra of a ^15^N-labelled tRNA_i_^Met^ measured in a time-resolved fashion during a continuous incubation at 30°C in yeast wild-type extract over 16 h. (**B**) Imino (^1^H,^15^N) correlation spectra of a ^15^N-labelled tRNA_i_^Met^ measured in a time-resolved fashion during a continuous incubation at 30°C in yeast *trm4Δ* extract over 16 h. Each NMR spectrum measurement spreads over a 2 h time period, as indicated.

The existence of a positive effect of m^5^Cs on m^1^A58 introduction in tRNA_i_^Met^*in vitro* (Figure 3B), does not necessarily imply that this effect occurs in a cellular context. For example, if m^1^A58 is introduced before m^5^Cs, no effect of m^5^Cs on the introduction of m^1^A58 can possibly be observed. In order to investigate whether this positive effect persists in a cellular context, we applied our recently developed methodology (39,40) to the monitoring of the introduction of m^1^A58 into tRNA_i_^Met^ in yeast extracts. As seen above, the imino signals of G18 and U55 constitute an NMR signature of a properly assembled elbow structure, and therefore can be regarded as an indirect marker of m^1^A58 introduction in the case of tRNA_i_^Met^. We made use of this marker to monitor the introduction of m^1^A58 in tRNA_i_^Met^ in wild-type and in *trm4Δ* yeast extracts using time-resolved NMR. For that, ^15^N-labelled unmodified tRNA_i_^Met^ was incubated at 30°C in yeast extracts supplemented with the modification enzymes cofactors, SAM and NADPH. A series of ^1^H-^15^N BEST-TROSY experiments were measured for wild-type and *trm4Δ* yeast extracts (Figure 6). The observation of the imino signals of G18 and U55 along the tRNA_i_^Met^ maturation routes revealed that m^1^A58 is introduced slightly faster in the wild-type extract than in an extract depleted of Trm4. This shows that lack of m^5^C48,49 has a negative effect on m^1^A58 introduction by Trm6/Trm61 in yeast tRNA_i_^Met^, which is perfectly consistent with the *in vitro* kinetic assays on tRNA_i_^Met^ (Figure 3B). Overall, our data show that m^5^C modifications have a positive effect on m^1^A58 introduction in tRNA_i_^Met^ both *in vitro* and in a cellular context.

## DISCUSSION

In this study, we implemented a generic approach for the preparation of specifically modified tRNAs in order to pursue a thorough investigation of the cross-talk between modifications Ψ55, T54 and m^1^A58 in yeast tRNA^Phe^. We demonstrated a direct positive and cumulative effect of modifications Ψ55 and T54 on the incorporation of m^1^A58 in this elongator tRNA. Conversely, we report that m^1^A58 is efficiently introduced on unmodified initiator tRNA_i_^Met^ without the need of any prior modification, revealing distinct pathways for m^1^A58 incorporation in yeast elongator and initiator tRNAs. Finally, we show that the m^1^A58 single modification has a considerable impact on the structural properties of yeast tRNA_i_^Met^. This provides an explanation with structural basis for the degradation of hypomodified tRNA_i_^Met^ lacking m^1^A58 by the nuclear surveillance and RTD pathways. Our study has important implications for understanding tRNA modification pathways and in particular for the investigation of modification circuits. These aspects are discussed below.

Genetic approaches are very effective strategies for identifying cross-talk between different genes, and genes encoding modification enzymes are no exception (30). These are however most effective when used in conjunction with biochemical approaches, allowing for a detailed characterization of the molecular aspects contributing to the observed phenotypes. Using specific deletion strains, we previously identified an interdependence between the Ψ55, T54 and m^1^A58 modifications in yeast tRNA^Phe^ from the observation of a slow-down in the incorporation of certain modifications in the absence of other specific enzymes (39). With a biochemical approach, we now establish that the incorporation of T54 is directly stimulated by Ψ55, and that the incorporation of m^1^A58 is directly and individually stimulated by Ψ55 and T54, with a notable cumulative effect when they are both present, thus reporting that the effects of the modifications are direct and not the result of other indirect effects. These modification circuits in the T-arm of yeast elongator tRNAs concern modifications T54, Ψ55 and m^1^A58, which are among the most conserved modified nucleotides in all sequenced tRNAs (58,59). These modifications participate in maintaining the universal tRNA tertiary fold, more precisely at the level of the elbow region, assembled via conserved contacts between the T- and D-loops (57,60). The characterization of this circuit involving modifications of the tRNA core is therefore of general interest for understanding the relation between modifications and structure in tRNAs.

Simple chemical modifications, namely an isomerisation in case of Ψ55, and a methylation in case of T54, can thus render a given tRNA a substantially better substrate for subsequent modification enzymes. The Ψ55 $ \to$ T54 $ \to$ m^1^A58 and Ψ55 $ \to$ m^1^A58 modification circuits reported here are robust circuits with highly pronounced effects, with for instance an initial velocity of m^1^A58 incorporation that is increased by a factor 15 in presence of both Ψ55 and T54 (Table 2). The presence of Ψ55 alone also greatly stimulates the activity of Trm6/Trm61, with a positive effect on m^1^A58 incorporation that is about two-times larger than the positive effect of T54 (Table 2). This marked effect of Ψ55 leads to undetectable levels of m^1^A58 along the maturation route of tRNA^Phe^ in *pus4Δ* yeast extracts monitored by NMR spectroscopy (39). In addition, previous time-resolved NMR study of tRNA^Phe^ in *pus4Δ* and *trm2Δ* yeast extracts pointed towards the m^1^A58 incorporation being more affected by Ψ55 than by T54, which is perfectly in agreement with the kinetic data reported here. This indicates that the time-resolved NMR approach we have developed in cellular extracts (40), is not only reliable to identify cross-talks between modifications, but also to discriminate between weak and strong dependencies.

The question remains of the molecular origin of the differences in the catalytic efficiencies of Trm6/Trm61 regarding tRNA^Phe^ and tRNA_i_^Met^, as well as of the molecular basis of such ordered modification circuit in tRNA^Phe^. In a circuit of modifications, the observed effect of the initial modification on the subsequent enzyme is reflected in an increased turnover rate, meaning either a better substrate binding, or a better catalytic efficiency, or a better product release, depending on the enzyme considered (61). For RNA modification enzymes, the rate-determining step of the reaction has been reported to be the catalytic step (62,63), the product release (64,65), or conformational changes of both the RNA and protein, most probably to accommodate the target nucleotide into the active site (66). Since yeast Trm6/Trm61 exhibits high structural similarity with its human homolog (67), the structure of human Trm6/Trm61 in complex with tRNA^Lys(UUU)^ can be examined to understand the tRNA recognition and modification mechanism of Trm6/Trm61 (68). This structure reveals that unfolding of the tRNA tertiary structure is required to allow access to the methylation target A58. In particular, the interactions between the T- and D-loops are disrupted and the D-arm is moved away from its position as a result of interactions with the N-terminal β-barrel domain of Trm61. Nucleotides 55–60 in the T-loop also change their conformation to accommodate the A58 target into the Trm61 active site (68). This structure suggests that a weak interaction between the D- and T-arms would probably lead to a more favorable substrate accommodation for m^1^A58 modification by Trm6/Trm61. This could explain why unmodified tRNA_i_^Met^, where the tRNA elbow is not properly assembled without the m^1^A58 modification (Figure 5), is efficiently modified by Trm6/Trm61. On the contrary, given the structural properties of the unmodified tRNA^Phe^ (Supplementary Figure S6), this substrate would be less favourably recognized by Trm6/Trm61. Furthermore, in the case of elongator tRNA^Phe^, the stabilization of the T-arm structure via the modifications T54 and Ψ55 has a positive effect on m^1^A58 incorporation by Trm6/Trm61 (Figure 2 and Table 1). The comparison of the NMR spectra of Ψ55- and T54-containing tRNA^Phe^ with that of the unmodified tRNA^Phe^, shows very limited chemical shift variations, suggesting that these modifications do not induce large global rearrangements in the structure of tRNA^Phe^ (39,49). However, since nucleotides 55–60 in the T-loop largely change their conformation upon accommodation of the A58 target into the active site of Trm61 (68), modifications Ψ55 and T54 may directly affect this step of T-loop reorganization. These modifications could indeed lead to local and/or global changes in the dynamic properties of the tRNA substrate. Such a mechanism has recently been reported in the case of *E. coli* tRNA_f_^Met^, in which conformational fluctuations on the local level are increased in the modified tRNA (69). Modifications could thus help reach otherwise inaccessible structural conformations that are more suited to substrate accommodation by the next modification enzyme, which would explain the increased efficiency of m^1^A58 incorporation in presence of T54 and Ψ55.

Even though modification circuits are widespread and have been reported in several organisms, including *S. cerevisiae*, *S. pombe*, *E. coli*, *T. thermophilus*, drosophila, human and plants (32–38,70–74), the role of such ordered circuits of modifications remains an open question. For modification circuits in the anticodon-loop region, however, it has been recently proposed that modifications introduced first act as additional recognition elements for the subsequent enzyme. This would provide the means for adding modifications with considerable variation in the anticodon-loop region (31). This hypothesis is quite convincing for modifications in the anticodon-loop region, but cannot explain the actual modification circuit in the T-loop of yeast elongator tRNAs. This modification circuit in the tRNA core indeed involves modifications that are highly conserved. Until recently, modification circuits in the tRNA core region have been only reported in the case of the extremely-thermophilic bacterium *T. thermophilus* (37,38), and are most likely not implicated in sequential orders of modification incorporation, but rather in a fine tuning of modification levels in relation to an adaptation to variations in growth temperature (75). The Ψ55 $ \to$ T54 $ \to$ m^1^A58 modification circuit in yeast elongator tRNAs therefore constitutes the first description of an ordered circuit of modification involving modifications from the tRNA core region. Since their identification remains difficult, particularly because real-time monitoring of tRNA maturation at a single nucleotide level is technically challenging (76), we are convinced that modification circuits in the tRNA core region are certainly more widespread than currently thought. Such circuits are likely to be identified in the near future through the use of nanopore sequencing technologies applied to tRNAs (77,78).

One of the most striking features of our study concerns the changes in the structural properties of tRNA_i_^Met^ upon m^1^A58 modification. NMR-fingerprints of unmodified tRNA_i_^Met^ and m^1^A58-tRNA_i_^Met^ indeed revealed important structural rearrangements upon addition of a single methyl group. Even though the NMR spectra of unmodified tRNA_i_^Met^ indicate a certain dynamic that probably leads to intermediate exchange on the NMR chemical shift time scale, the presence at the almost exact same chemical shifts of the imino groups of U42, U50, G68, G70, G12, G24 and G30 in the NMR-fingerprints of unmodified- and m^1^A58-tRNA_i_^Met^ attest to the proper secondary structure assembly of this tRNA (Figure 5). All RNA helices, namely the T-, D-, anticodon- and the acceptor-stems, are thus likely correctly assembled. However, the three-dimensional structure of the tRNA is not properly formed as demonstrated by the lack of signals attesting to a properly assembled tRNA elbow structure, namely imino groups of U55 and G18 (Figure 5). These structural rearrangements of yeast tRNA_i_^Met^ upon m^1^A58 modification are most probably at the origin of the specific degradation of hypomodified tRNA_i_^Met^ lacking m^1^A58 by the nuclear surveillance and RTD pathways (27,28,41,42), while the properly folded m^1^A58-tRNA_i_^Met^ is protected from degradation. It is noteworthy that other hypomodified tRNAs lacking at least one tRNA core modification are targeted to degradation by the RTD pathway. In all reported cases, this degradation in the absence of one or two modifications is tRNA specific, meaning that only specific tRNAs are targeted to degradation. For instance, tRNA^Val(AAC)^ lacking m^7^G46 and m^5^C49 is rapidly degraded by the RTD pathway in *S. cerevisiae* (24); tRNA^Ser(CGA)^ and tRNA^Ser(UGA)^ lacking either Um44 and ac^4^C12 or m^2,2^G26 and m^5^C48 are also rapidly degraded by the RTD pathway in *S. cerevisiae* (26,79); tRNA^Tyr(GUA)^ and tRNA^Pro(AGG)^ lacking m^7^G46 are rapidly degraded by the RTD pathway in *S. pombe* (80). Comparing these reports with the case of tRNA_i_^Met^ lacking m^1^A58, it is tempting to speculate that the modifications involved might be responsible for large structural effects and stabilize the tRNA tertiary structure in these particular cases. The same modifications would, in comparison, not much alter much the structure of non-targeted tRNAs, a hypothesis that would need to be tested experimentally in future structural work.

Another important point revealed by the monitoring of the m^1^A58 introduction in tRNA_i_^Met^ in yeast extracts resides in the fact that our NMR-based methodology for monitoring tRNA maturation in cell extracts has the ability to report both on the introduction of chemical modifications, and on structural changes occurring during maturation. This point was not fully appreciated in the NMR study of yeast tRNA^Phe^, since this tRNA is, to a certain extent, properly folded without modifications (39). Changes in the NMR spectra of tRNA^Phe^ upon modification are modest (39,49), and mainly reflect the incorporation of new chemical groups, with probably also some minor structural rearrangements. The example of tRNA_i_^Met^ has highlighted that NMR spectroscopy is an ideal method that can report, in a time-resolved fashion, on how the modification process affects tRNA structural properties.

In this work, we have described different modification pathways for m^1^A58 incorporation in yeast elongator and initiator tRNAs. Unmodified elongator tRNA^Phe^ is an intrinsically poor substrate of Trm6/Trm61, whereas unmodified tRNA_i_^Met^ is an intrinsically good substrate of the same enzyme. This raises the general question of what makes a good versus a poor substrate for a modification enzyme? To look into this matter, it is important to bear in mind that modifications may not necessarily have the same beneficial effect on all tRNAs (81). For instance, a certain modification may be particularly important for a certain tRNA, which constitutes the evolutionary pressure for retaining this modification enzyme, but might be much less important, if significant at all, in other tRNAs. In this context, dealing with good and poor substrates represents an ordinary challenge faced by modification enzymes. Indeed, tRNAs are to some extent sufficiently similar to be recognized and employed by the translation machinery, but need at the same time to be sufficiently different to be uniquely recognized by their cognate aminoacyl-tRNA synthetases. The modification enzymes therefore should handle a population of highly similar but unique tRNAs and the tRNA modification patterns can be regarded as the result of millions of years of coevolution of modification enzymes with the tRNA population (11,82). In this context, we believe that a potential role of modification circuits could be to allow the modification of both good and poor tRNA substrates. In the case of yeast elongator and initiator tRNAs, which must have sufficiently different structural properties to be recognized by elongation or initiation factors, the existence of the Ψ55 $ \to$ T54 $ \to$ m^1^A58 modification circuit enables the incorporation of m^1^A58 in certain elongator tRNAs, such as tRNA^Phe^, which are poor intrinsic substrate of Trm6/Trm61. In conclusion, modification circuits might be a solution found to deal with the problem of having at the same time poor and good tRNA substrates that all require to be eventually modified.

## Supplementary Material

gkad722_Supplemental_File

## Data Availability

The data underlying this article will be shared on reasonable request to the corresponding author.
