# Supplementary material for: Different modification pathways for m1A58 incorporation in yeast elongator and initiator tRNAs
Source: Nucleic Acids Res. 2023 Aug 31;51(19):10653–67. doi: 10.1093/nar/gkad722 (PMC10602860; doi:10.1093/nar/gkad722)
Supplement: gkad722_Supplemental_File [file gkad722_supplemental_file.pdf]

# Supplementary Material

## Different modification pathways for m$^1$A58 incorporation in yeast elongator and initiator tRNAs

Marcel-Joseph Yared, Yasemin Yoluç, Marjorie Catala, Carine Tisné, Stefanie Kaiser & Pierre Barraud

---

Content:

Supplementary Tables S1 to S4 pages 2-6
Supplementary Figures S1 to S7 pages 7-13
Supplementary References page 14

## SUPPLEMENTARY TABLES

### Supplementary Table S1 – Yeast and *E. coli* strains used in this study

| Strain | Genotype | Source |
|---|---|---|
| BY4741 | *MATa his3-Δ1 leu2-Δ0 met15-Δ0 ura3-Δ0* | Euroscarf |
| BY4741-*dus1Δ* | BY4741, *YML080w::kanMX* | Euroscarf |
| BY4741-*dus3Δ* | BY4741, YLR401c*::kanMX* | Euroscarf |
| BY4741-*pus4Δ* | BY4741, *YNL292w::kanMX* | Euroscarf |
| BY4741-*trm1Δ* | BY4741, *YDR120c::kanMX* | Euroscarf |
| BY4741-*trm2Δ* | BY4741, *YKR056w::kanMX* | Euroscarf |
| BY4741-*trm4Δ* | BY4741, *YBL024w::kanMX* | Euroscarf |
| BY4741-*trm8Δ* | BY4741, *YDL201w::kanMX* | Euroscarf |
| BY4741-*trm10Δ* | BY4741, *YOL093w::kanMX* | Euroscarf |
| BY4741-*trm11Δ* | BY4741, *YOL124c::kanMX* | Euroscarf |
| BY4741-*rit1Δ* | BY4741, *YMR283c::kanMX* | Euroscarf |
| c13-ABYS-86 | *MATα ura3Δ5 leu2-3,112 his3 pra1-1 prb1-1 prc1-1 cps1-3* | (1) |
| c13-ABYS-86-*trm4Δ* | c13-ABYS-86, *YBL024w::kanMX* | (2) |
| | | |
| BW25113-*ygghΔ* | *E. coli* K-12 $lacI^{+}rrnB_{T14}$ $\Delta lacZ_{WJ16}$ *hsdR*514 $\Delta araBAD_{AH33}$ $\Delta rhaBAD_{LD78}$ *rph-1* Δ*(araB–D)567* Δ*(rhaD–B)568* Δ*lacZ4787*(::*rrnB-3*) *hsdR514 rph-1 yggH::kan* | (3) |
| BL21-CodonPlus (DE3)-RIL | *E. coli* B $F^{-}$ ompT hsdS($r_B^{-}m_B^{-}$) $dcm^{+}$ $Tet^{r}$ gal λ(DE3) endA Hte [argU ileY leuW $Cam^{r}$] | Agilent |
| BL21-CodonPlus (DE3)-RIL-*yggHΔ* | *E. coli* BL21-CodonPlus(DE3)-RIL *yggH::kan* | This study |

**Supplementary Table S2 – List of primers used in this study**

Pus4-M1_EcoRI_Fwd: CAG-GCG-AAT-TCA-TGA-ATG-GAA-TAT-TTG-CTA-TT
Pus4-V403_NotI_Rev: TAC-AAG-GCG-GCC-GCT-TAC-ACC-TGT-TCG-ATT-TT

Trm2-M1_EcoRI_Fwd: GAC-AAA-GAA-TTC-ATG-TAC-GAA-CAG-TTT-GAA-TTT
Trm2-I639_NotI_Rev: TAT-TTT-GCG-GCC-GCA-ATT-AGA-TTC-TCT-TCA-TTA
Trm2_BamHI-del_Fwd: GTT-ATC-TTG-GAC-CCA-CCA-CGC-AAG-GGC-TGT-GAC-GAA-TTA-TTC
Trm2_BamHI-del_Rev: TGG-TGG-GTC-CAA-GAT-AAC-GGA-AGT-GTT-TTC-ACT-TGG-AGT-ATC-AAT-AG
Trm2-V116_BamHI_Fwd: CGA-TGG-ATC-CGT-TGA-AAC-AAC-TTC-TCC-GAT-GG
Trm2-I639_XhoI_Rev: GCG-CCT-CGA-GTT-AGA-TTC-TCT-TCA-TTA-TAC-ACA-C

Trm6-M1_BamHI_Fwd: AAT-TTA-GGA-TCC-GAT-GAA-TGC-TTT-GAC-AAC
Trm6-I478_NotI_Rev: CTC-TGC-GGC-CGC-TTA-TAT-CTT-TTG-TTT-CTT-AG
Trm61-M1_NdeI_Fwd: TTT-ACA-TAT-GTC-AAC-AAA-TTG-TTT-TTC-CGG-TTA
Trm61-K383_XhoI_Rev: TAA-TCC-TCG-AGT-TAT-TTT-TCC-GTG-GAT-CGA-AGA

yggH_A: GGT-GCG-TAC-CTC-ATC-CAG-TT
yggH_B: GTA-TCG-TTC-AGG-TGC-CGT-TT
yggH_C: CTG-CTC-AGG-GCG-ATC-TTT-AG
yggH_D: CGC-CAT-AAA-GCG-TTC-AAA-AT

k1: CAG-TCA-TAG-CCG-AAT-AGC-CT
k2: CGG-TGC-CCT-GAA-TGA-ACT-GC

rit1_C: TCT-AGG-AAA-AGT-GAG-TTC-AGG-CTT-A
rit1_D: TCG-TGT-AAA-ACC-TCA-GAA-CAC-TGT-A

dus3_C: GGT-CTA-GGC-AAC-AAA-GGT-ACA-CTA-A
dus3_D: TAT-TTT-GAT-TTT-CTT-GGA-ACC-CAT-A

trm10_C: TAC-CAA-TTA-TGA-AAA-CTG-GAA-CCA-T
trm10_D: TAC-AAC-ATC-AAA-GCA-AAA-TAA-GCA-A

kanC: TGA-TTT-TGA-TGA-CGA-GCG-TAA-T

T7 promoter: 5'TAA-TAC-GAC-TCA-CTA-TAG 3'

yeast $tRNA^{Phe}$-WT DNA template :
3' ATTATGCTGAGTGATATCGCCTAAATCGAGTCAACCCTCTCGCGGTCTGACTTCTAGACCTCCAGGACACAAGCTAGGTGTCTTAAGCGTGGT 5'
yeast $tRNA^{Phe}$-ΔU17 DNA template:
3' ATTATGCTGAGTGATATCGCCTAAATCGAGTCACCCTCTCGCGGTCTGACTTCTAGACCTCCAGGACACAAGCTAGGTGTCTTAAGCGTGGT 5'
yeast $tRNA^{Phe}$-A20A60 DNA template:
3' ATTATGCTGAGTGATATCGCCTAAATCGAGTCAACCTTCTCGCGGTCTGACTTCTAGACCTCCAGGACACAAGCTATGTGTCTTAAGCGTGGT 5'
yeast $tRNA^{Phe}$-A54 DNA template:
3' ATTATGCTGAGTGATATCGCCTAAATCGAGTCAACCCTCTCGCGGTCTGACTTCTAGACCTCCAGGACACTAGCTAGGTGTCTTAAGCGTGGT 5'
yeast $tRNA^{Phe}$-UAAA DNA template:
3' ATTATGCTGAGTGATATCGCCTAAATCGAGTCACCTTCTCGCGGTCTGACTTCTAGACCTCCAGGACACTAGCTATGTGTCTTAAGCGTGGT 5'

yeast $tRNA_i^{Met}$-WT DNA template:
3′ ATTATGCTGAGTGATATCCGCGGCACCGCGTCACCTTCGCGCGTCCCGAGTATTGGGACTACAGG
AGCCTAGCTTTGGCTCGCCGCGGTGGT 5′
yeast $tRNA_i^{Met}$-U17 DNA template:
3′ ATTATGCTGAGTGATATCCGCGGCACCGCGTCAACCTTCGCGCGTCCCGAGTATTGGGACTACAG
GAGCCTAGCTTTGGCTCGCCGCGGTGGT 5′
yeast $tRNA_i^{Met}$-G20C60 DNA template:
3′ ATTATGCTGAGTGATATCCGCGGCACCGCGTCACCCTCGCGCGTCCCGAGTATTGGGACTACAGG
AGCCTAGCTTGGGCTCGCCGCGGTGGT 5′
yeast $tRNA_i^{Met}$-U54 DNA template:
3′ ATTATGCTGAGTGATATCCGCGGCACCGCGTCACCTTCGCGCGTCCCGAGTATTGGGACTACAGG
AGCCAAGCTTTGGCTCGCCGCGGTGGT 5′
yeast $tRNA_i^{Met}$-UGCU DNA template:
3′ ATTATGCTGAGTGATATCCGCGGCACCGCGTCAACCCTCGCGCGTCCCGAGTATTGGGACTACAG
GAGCCAAGCTTGGGCTCGCCGCGGTGGT 5′
*Remark: The A1-U72 base pair was replaced with a G1-C72 base pair to improve in vitro transcription efficiency for all $tRNA_i^{Met}$ variants.*

reverse complementary oligo for yeast $tRNA_i^{Met}$ purification:
5′ [Biotin]-AAA-TCG-GTT-TCG-ATC-CGA-GGA-CAT-CAG-GGT-TAT-GA 3′

**Supplementary Table S3 – Purification conditions of the modification enzymes used in this study**

| | Pus4 | Trm2 | Trm6/Trm61 | Trm4 |
|---|---|---|---|---|
| Cooled down at | 30°C | 25°C | 18°C | 30°C |
| IPTG induction concentration | 0.5 mM | 0.5 mM | 0.4 mM | 0.5 mM |
| Growing time after induction | 6 h | 6 h | 22 h | 17 h |
| Lysis Buffer | 50 mM Tris-HCl pH 8.0, 300 mM NaCl, 10 mM BME, 1 mM PMSF, 10% (v/v) glycerol, 1 mM EDTA | 50 mM Tris-HCl pH 8.0, 1 M NaCl, 5 mM $MgCl_2$, 1 mM DTT, 5% (v/v) glycerol, 1 mM EDTA | 50 mM Tris-HCl pH 8.0, 300 mM NaCl, 1 mM PMSF, 1 mM DTT, 5% (v/v) glycerol, 1 mM EDTA | 50 mM Tris-HCl pH 8.0, 0.5 M NaCl, 1 mM DTT, 0.1% (v/v) Triton X-100, 1 mM EDTA |
| Purification steps | -HisTrap HP<br>-Superdex 75 | -HisTrap HP<br>-HiPrep Phenyl HP<br>-Superdex 75 | -HisTrap HP<br>-Superdex 200 | -HisTrap HP<br>-Superdex 200 |
| Storage Buffer | 50 mM Tris-HCl pH 8.0, 150 mM NaCl, 2 mM BME | 50 mM Tris-HCl pH 8.0, 400 mM NaCl, 1 mM DTT | 50 mM Tris-HCl pH 8.0, 300 mM NaCl, 5 mM DTT | 50 mM Tris-HCl pH 8.0, 300 mM NaCl, 1 mM DTT, 5% (v/v) glycerol |
| Concentration | to ~10 mg/mL with Amicon 10,000 MWCO | to ~5 mg/mL with Amicon 50,000 MWCO | to ~5 mg/mL with Amicon 50,000 MWCO | to ~8 mg/mL with Amicon 50,000 MWCO |
| mM extinction coefficient | 30.4 $mM^{-1}.cm^{-1}$ | 30.8 $mM^{-1}.cm^{-1}$ | 89 $mM^{-1}.cm^{-1}$ | 80.9 $mM^{-1}.cm^{-1}$ |
| Vector | pET28-Pus4 | pSUMO-Trm2 | pETDuet-Trm6/Trm61 | pET28-Trm4 |
| Expression strain | *E. coli* BL21(DE3) CodonPlus-RIL | *E. coli* BL21(DE3) CodonPlus-RIL | *E. coli* BL21(DE3) CodonPlus-RIL *yggH::kan* | *E. coli* BL21(DE3) CodonPlus-RIL |
| Purification adapted from reference | (4) | - | - | (5) |

IPTG: isopropyl-β-D-thiogalactopyranoside; BME : 2-mercaptoethanol; PMSF: phenylmethanesulfonylfluoride; EDTA: ethylenediaminetetraacetic acid; DTT: dithiothreitol

**Supplementary Table S4**

| Enzyme | Trm2 | | Trm6/Trm61 | | | | | |
|---|---|---|---|---|---|---|---|---|
| tRNA quantity | 500 pmol | | 500 pmol | | | | | |
| Yeast tRNAs | unmodified - $tRNA^{Phe}$ | Ψ55-$tRNA^{Phe}$ | unmodified - $tRNA^{Phe}$ | T54-$tRNA^{Phe}$ | Ψ55-$tRNA^{Phe}$ | Ψ55-T54-$tRNA^{Phe}$ | unmodified-$tRNA_i^{Met}$ | $m^5$C48,49-$tRNA_i^{Met}$ |
| Enzyme (pmol) | 20 | 2.5 | 15 | 15 | 5 | 2.5 | 7.5 | 7.5 |
| SAM (pmol) | 880 | 880 | 880 | 880 | 880 | 880 | 880 | 880 |
| [$^3$H]-SAM (pmol) | 2.5 | 2.5 | 2.5 | 2.5 | 2.5 | 2.5 | 2.5 | 2.5 |
| Reaction conditions | MB1X 30°C 30 min | MB1X 30°C 30 min | MB1X 30°C 96 min | MB1X 30°C 30 min | MB1X 30°C 30 min | MB1X 30°C 30 min | MB1X 30°C 30 min | MB1X 30°C 30 min |

The different kinetic reaction mixes and conditions to determine the initial velocities of Trm2 introducing the T54 modification and Trm6/Trm61 introducing the $m^1$A58 modification to a set of specifically modified yeast tRNAs. MB corresponds the maturation buffer: 100 mM $NaH_2PO_4/K_2HPO_4$ pH 7.0, 5 mM $NH_4Cl$, 2 mM DTT and 0.1 mM EDTA. These quantities correspond to a volume of 50 µL of reaction.

## SUPPLEMENTARY FIGURES

a

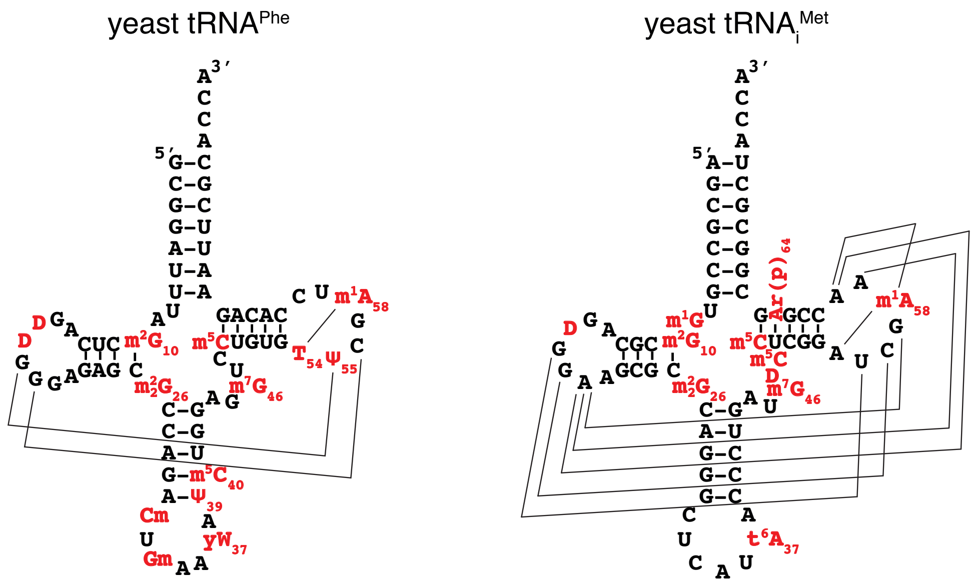


b

| Modification | Enzyme | Partner protein |
|---|---|---|
| m$^2$G10 | Trm11 | Trm112 |
| D16 | Dus1 | - |
| D17 | Dus1 | - |
| m$^2_2$G26 | Trm1 | - |
| Cm32 | Trm7 | Trm732 |
| Gm34 | Trm7 | Trm734 |
| yW37 | Trm5, TYW1-4 | - |
| Ψ39 | Pus3 | - |
| m$^5$C40 | Trm4 | - |
| m$^7$G46 | Trm8 | Trm82 |
| m$^5$C49 | Trm4 | - |
| m$^5$U54 (T54) | Trm2 | - |
| Ψ55 | Pus4 | - |
| m$^1$A58 | Trm61 | Trm6 |

| Modification | Enzyme | Partner protein |
|---|---|---|
| m$^1$G9 | Trm10 | - |
| m$^2$G10 | Trm11 | Trm112 |
| D16 | Dus1 | - |
| m$^2_2$G26 | Trm1 | - |
| t$^6$A37 | Sua5 | EKC/KEOPS |
| m$^7$G46 | Trm8 | Trm82 |
| D47 | Dus3 | - |
| m$^5$C48 | Trm4 | - |
| m$^5$C49 | Trm4 | - |
| m$^1$A58 | Trm61 | Trm6 |
| Ar(p)64 | Rit1 | - |

**Supplementary Figure S1: Sequence and modifications of yeast elongator tRNA$^{Phe}$ and yeast initiator tRNA$_i^{Met}$**

(**a**) Sequence and cloverleaf representation of yeast tRNA$^{Phe}$ (*left*) and yeast tRNA$_i^{Met}$ (*right*). Main tertiary interaction between the D- and T-loops are represented with thin lines. Modifications are highlighted in red. (**b**) Table of correspondence between the modifications and the enzyme responsible for their introduction in yeast tRNA$^{Phe}$ (*left*) and in yeast tRNA$_i^{Met}$ (*right*).

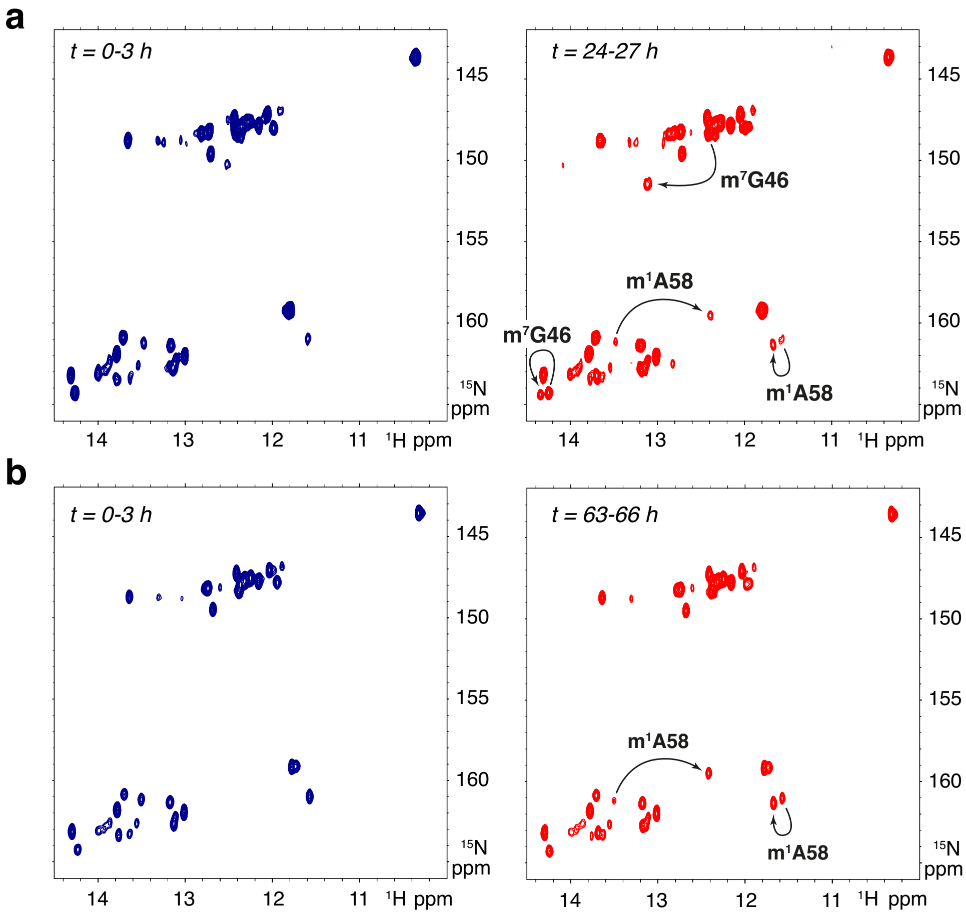


**Supplementary Figure S2: Contamination of Trm6/Trm61 with m$^{7}$G46 activity**

(**a**) ($^{1}$H,$^{15}$N)-BEST-TROSY experiments on $^{15}$N-labelled tRNA$^{Phe}$ reporting on Trm6/Trm61 enzymatic activity purified from *E. coli* BL21(DE3)CodonPlus-RIL cells. Concentrations: tRNA$^{Phe}$ at 100 µM and Trm6/Trm61 at 2 µM. Although the m$^{1}$A58 activity is clearly observable, the Trm6/Trm61 sample is contaminated with m$^{7}$G46 activity. (**b**) ($^{1}$H,$^{15}$N)-BEST-TROSY experiments on $^{15}$N-labelled tRNA$^{Phe}$ reporting on Trm6/Trm61 enzymatic activity purified from *E. coli* BL21(DE3)CodonPlus-RIL *yggh::kan* cells. The sample is no longer contaminated with m$^{7}$G46 activity.

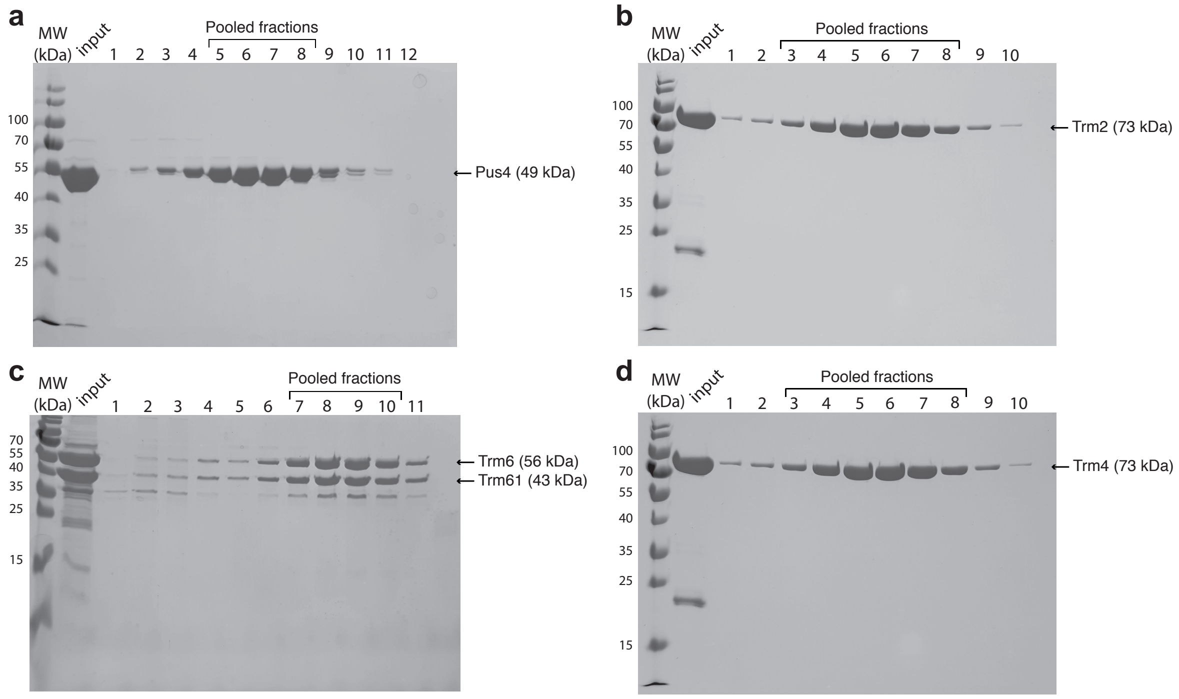


**Supplementary Figure S3: Quality control in the last step of purification of the modification enzymes**

(**a**) SDS-PAGE of the fractions of the Superdex 75 size exclusion column of Pus4 purification. (**b**) SDS-PAGE of the fractions of the Superdex 200 size exclusion column of Trm2 purification. (**c**) SDS-PAGE of the fractions of the Superdex 200 size exclusion column of Trm6/Trm61 purification. (**d**) SDS-PAGE of the fractions of the Superdex 200 size exclusion column of Trm4 purification. (MW): protein ladder, size in kDa. (input): fraction injected onto the column. For each purification, the pooled fractions are indicated above the gel.

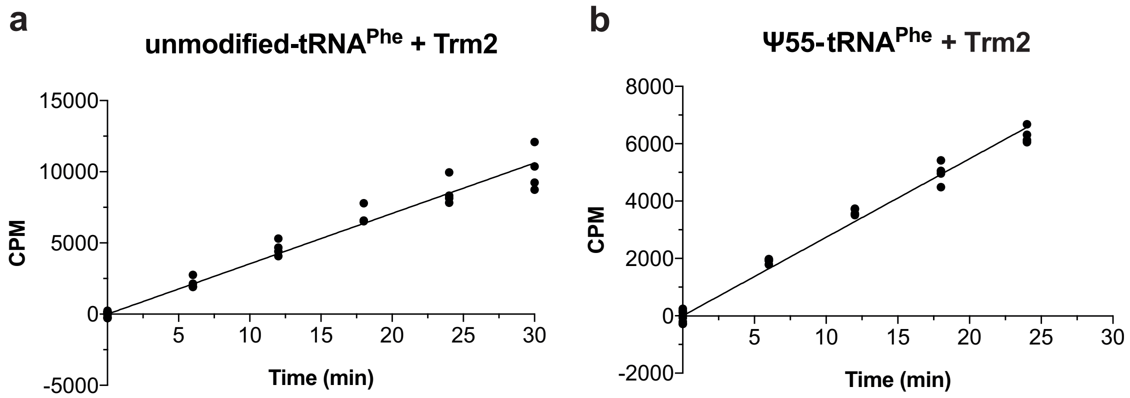


**Supplementary Figure S4: Kinetic efficiency of Trm2 depending on the modification profiles of yeast $tRNA^{Phe}$**

(**a**) Raw counts per minute (CPM) data corresponding to T54 introduction by Trm2 on the yeast unmodified-$tRNA^{Phe}$. (**b**) Raw CPM data corresponding to T54 introduction by Trm2 on the yeast $tRNA^{Phe}$ containing the Ψ55 modification (Ψ55-$tRNA^{Phe}$). CPM are measured for 4 or 5 time points in four independent experiments (N=4). Black dots represent individual measurements.

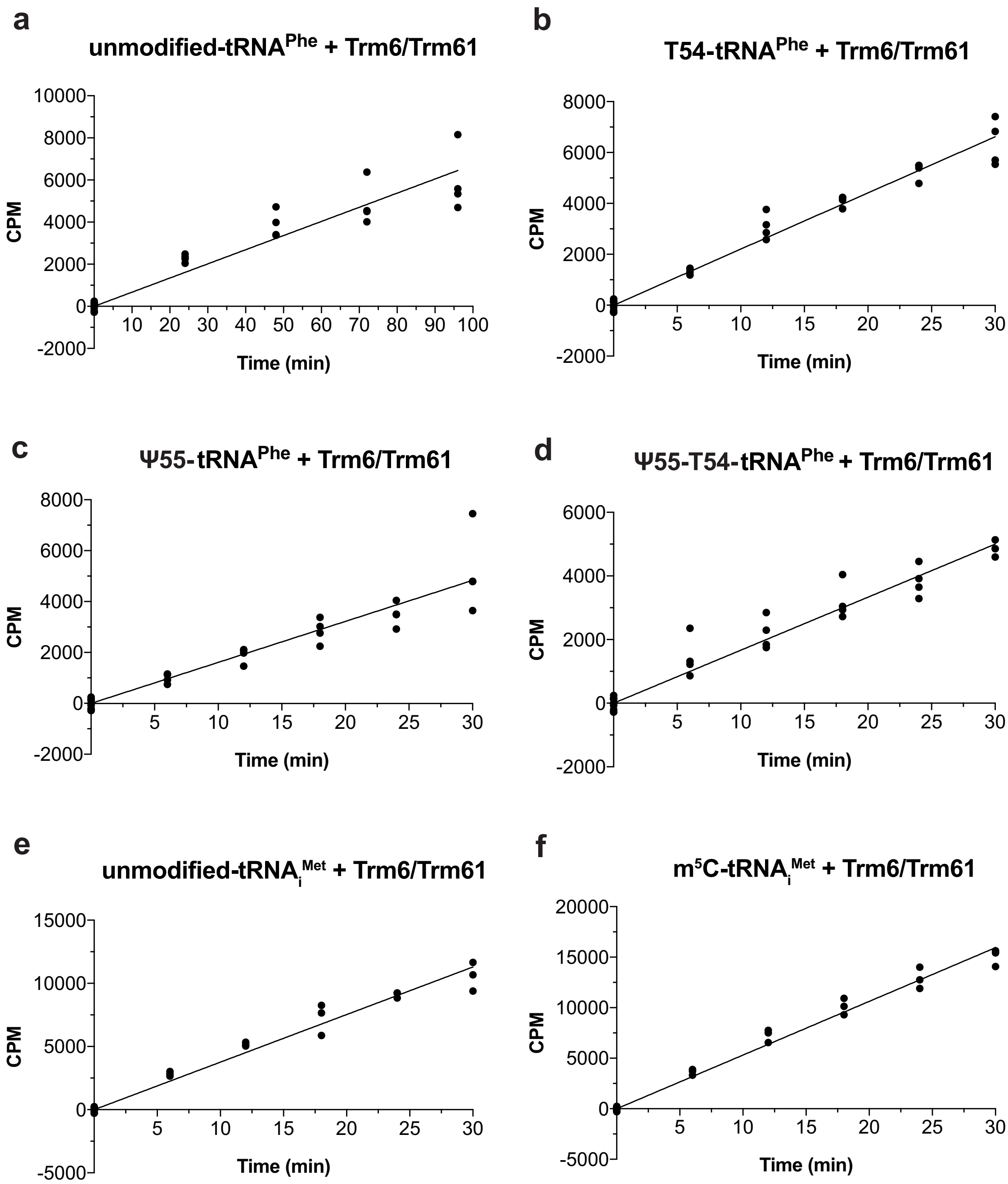


**Supplementary Figure S5: Kinetic efficiency of Trm6/Trm61 depending on the modification profiles of yeast tRNA$^{Phe}$ and tRNA$_i^{Met}$.**

(**a**) Raw counts per minute (CPM) data corresponding to the m$^1$A58 introduction by Trm6/Trm61 on the yeast unmodified-tRNA$^{Phe}$. (**b**) Raw CPM data corresponding to the m$^1$A58 introduction by Trm6/Trm61 on the tRNA$^{Phe}$ containing the T54 modification (T54-tRNA$^{Phe}$). (**c**) Raw CPM data corresponding to the m$^1$A58 introduction by Trm6/Trm61 on the tRNA$^{Phe}$ containing the Ψ55 modification (Ψ55-tRNA$^{Phe}$). (**d**) Raw CPM data corresponding to the m$^1$A58 introduction by Trm6/Trm61 on the tRNA$^{Phe}$ containing both the Ψ55 and T54 modifications (Ψ55-T54-tRNA$^{Phe}$). (**e**) Raw CPM data corresponding to the m$^1$A58 introduction by Trm6/Trm61 on the unmodified-tRNA$_i^{Met}$. (**f**) Raw CPM data corresponding to the m$^1$A58 introduction by Trm6/Trm61 on the tRNA$_i^{Met}$ containing the m$^5$C48,49 modifications (m$^5$C-tRNA$_i^{Met}$). CPM are measured for 4 or 5 time points in at least three independent experiments (N=3 or 4). Black dots represent individual measurements.

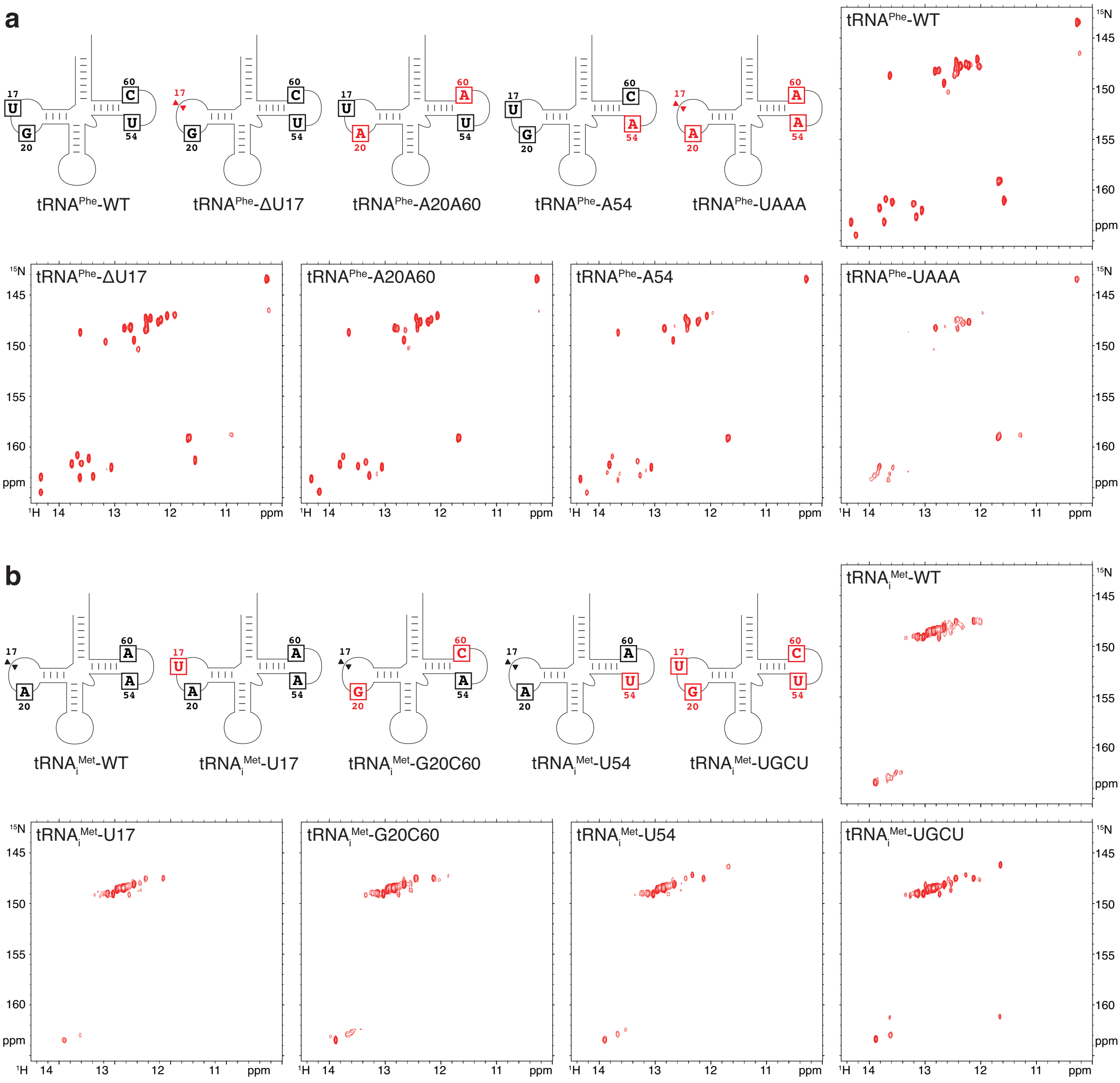


**Supplementary Figure S6: Effect of nucleotide mutations on the structural properties of yeast tRNA$^{Phe}$ and yeast tRNA$_i^{Met}$.**

(**a**) (*top left*) Schematic cloverleaf representation of yeast elongator tRNA$^{Phe}$ showing residues (boxed) characteristic of elongator tRNAs. Residues in black represent nucleotides that correspond to the WT sequence. Residues in red represent mutated nucleotides that correspond to the initiator tRNA$_i^{Met}$ sequence. The triangles in the D-loop denote that the U17 is absent. (*bottom right*) Imino ($^{1}$H,$^{15}$N) correlation spectra of $^{15}$N-labelled tRNA$^{Phe}$ WT and variants measured at 38°C in the NMR buffer.

(**b**) (*top left*) Schematic cloverleaf representation of yeast initiator tRNA$_i^{Met}$ showing residues (boxed) characteristic of initiator tRNA. Residues in black represent nucleotides that correspond to the WT sequence. Residues in red represent mutated nucleotides that correspond to the elongator tRNA$^{Phe}$ sequence. The triangles in the D-loop denote that the U17 is absent. (*bottom right*) Imino ($^{1}$H,$^{15}$N) correlation spectra of $^{15}$N-labelled tRNA$_i^{Met}$ WT and variants measured at 38°C in the NMR buffer.

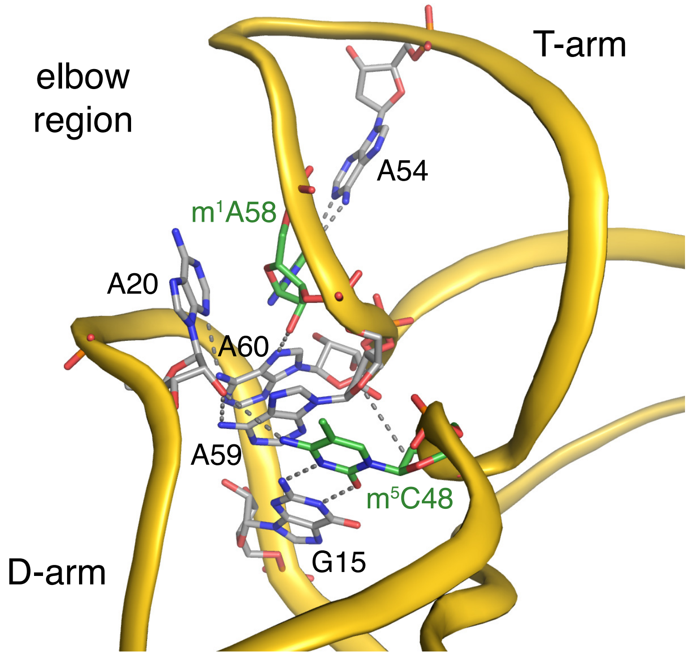


**Supplementary Figure S7: Close-up view of the intricate network of interaction at the level of the elbow region of initiator tRNA$_i^{Met}$**

Close-up view of yeast tRNA$_i^{Met}$ illustrating the proximity between nucleotides m$^5$C48 and m$^1$A58. The intricate network of interactions between nucleotides G15, m$^5$C48, A20, A59, A60, and m$^1$A58 is shown with potential hydrogen bonds as grey dashes. tRNA backbone is shown as a cartoon in yellow. Modified nucleotides m$^5$C48 and m$^1$A58 are shown in green.

## SUPPLEMENTARY REFERENCES


(1) Heinemeyer W, Kleinschmidt JA, Saidowsky J, Escher C, Wolf DH. Proteinase yscE, the yeast proteasome/multicatalytic-multifunctional proteinase: mutants unravel its function in stress induced proteolysis and uncover its necessity for cell survival. *EMBO J* (1991) 10:555-562.

(2) Barraud P, Gato A, Heiss M, Catala M, Kellner S, Tisné C. Time-resolved NMR monitoring of tRNA maturation. *Nat Commun* (2019) 10:3373.

(3) Baba T, Ara T, Hasegawa M, et al. Construction of Escherichia coli K-12 in-frame, single-gene knockout mutants: the Keio collection. *Mol Syst Biol* (2006) 2:2006.0008.

(4) Becker HF, Motorin Y, Planta RJ, Grosjean H. The yeast gene YNL292w encodes a pseudouridine synthase (Pus4) catalyzing the formation of psi55 in both mitochondrial and cytoplasmic tRNAs. *Nucleic Acids Res* (1997) 25:4493-99.

(5) Motorin Y, Grosjean H. Multisite-specific tRNA:m5C-methyltransferase (Trm4) in yeast Saccharomyces cerevisiae: identification of the gene and substrate specificity of the enzyme. *RNA* (1999) 5:1105-18.
